# SHINE study: Developing an intervention for safe hospital insulin use for older or frail adults with diabetes undergoing surgical hospital admission: Study protocol

**DOI:** 10.1371/journal.pone.0315387

**Published:** 2024-12-12

**Authors:** Christina Lange Ferreira, Sara Donetto, Hellena Habte-Asres, Jyothish Govindan, Angus Forbes, Kirsty Winkley

**Affiliations:** 1 Care in Long Term Conditions, Faculty of Nursing, Midwifery & Palliative Care, King’s College London, London, United Kingdom; 2 Diabetes and Endocrinology, Hereford County Hospital, Wye Valley NHS Trust, Hereford, United Kingdom; 3 Brighton and Sussex Medical School, Brighton, United Kingdom; Navrongo Health Research Centre, GHANA

## Abstract

**Aims:**

To present a study protocol for the development of an intervention to enhance safe insulin use for older or frail adults undergoing a surgical admission to hospital.

**Design:**

Following the United Kingdom’s Medical Research Council and National Institute for Health and Care Research Frameworks for development and evaluation of complex interventions; this qualitative study will use a co-design approach using design thinking, to develop a theoretical model for the intervention.

**Methods:**

Non-participatory observations, interviews and co-design workshops will be conducted with older or frail individuals with diabetes, their caregivers and healthcare staff responsible for their care during surgical admissions at a single National Health Service hospital in England. We will utilise their experiences and perspectives to establish priorities and generate ideas for the development of a conceptual model aimed at supporting the insulin safety review process in hospitals. Data will be analysed using framework analysis. People with diabetes were involved in the design of this study. The protocol was approved by the East-Midlands-Derby Research Ethics Committee (24/EM/0022). Study registered on Open Science Framework: https://osf.io/4wvu5.

**Results:**

Results of this study will be shared with study participants and disseminated through presentations at conferences/meetings and peer-reviewed publications.

**Conclusion:**

This article outlines the methodology for the planned study which will employ a novel methodology to tackle the problem of hospital insulin safety. Its findings will contribute to a better understanding of the multiple interacting components implicated in hospital insulin use (patient, staff, context) and support further work around system-based strategies to enhance insulin safety resilience in hospital.

## Introduction

Insulin is the primary treatment for Type 1 Diabetes and also required for many people living with Type 2 Diabetes [1, 2]. Insulin is considered a high-risk time critical medication, due to its narrow therapeutic index and level of harm that can occur in the event of an error: dangerously low or high glucose levels (hypoglycaemia or hyperglycaemia). Consequences of an error can range from mild to severe, including risk of death [3–5]. An estimated 237 million medication errors occur annually in the National Health Service (NHS), with associated costs upwards of 98 million pounds [6]. Errors in insulin use are frequent, occurring at all stages of the medication use process [5].

Following a scoping review, we identified significant variability in terms of exploring, defining, classifying and reporting insulin errors in hospital. To address this lack of consistency, we developed and used RESILIENT (inte**r**acting compon**e**nt**s** in **i**nsu**li**n us**e** in hospi**t**al) framework to analyse and map reported insulin errors in the included studies; identifying potential interacting components in insulin use (unpublished data). In this study we will extend this work by developing system-based learning models and resources to improve insulin safety.

### Background

In the United Kingdom (UK), one in six people occupying a hospital bed has diabetes [7]. People with diabetes (PWD) experience higher hospital length of stay (LOS) and readmission rates [8]. Hospital care environments are complex adaptive systems with multiple interacting components. Features such as self-organization; emergence, culture and gaps in the healthcare complex system, impact on the dynamic network of interactions between its multiple components, which can in turn lead to unpredictability and adaptations in behaviours of components [9]. In the context of insulin errors, multiple interacting factors within the hospital environment can increase risk of error. Risk factors can be observed at patient, health care professional (HCP) and systemic levels. Patient level factors include: impact of stress/illness on blood glucose levels; changes in appetite/ability to eat; altered insulin requirement; self-management capacity. HCP factors include: knowledge deficits (unfamiliarity with insulin types, regimens or technologies); poor communication (written or verbal); errors in documentation; high workloads and fatigue can have a negative impact on appropriate insulin administration, prescription and support [10, 11]. At a systemic level, factors such as changes in usual meal times, type of food available, new/changed treatments can contribute to the likelihood of an error [4].

Internationally, insulin has been identified as a high-risk medication in hospital environments; with frequent notifications received about insulin-related errors with adverse consequences reported [3, 10–12]. The UK National Diabetes Inpatient Audit (NADIA) highlighted 40% of insulin-treated inpatients experienced one or more insulin error [7]. Insulin use in hospital is a recognised source of anxiety and distress for PWD, who often report not feeling safe [8]. Whilst several national and local interventions/initiatives have been designed to improve insulin safety in UK hospitals [7, 8, 12], the problem remains, with variable performance in diabetes care across NHS hospitals [12]. This study will aim to address this problem by bringing together NHS staff, people with diabetes and their family members/carers, to co-design a multimodal intervention to target underpinning factors that can contribute to insulin errors.

#### Diabetes and perioperative care

Minimising hyperglycaemia prior to, during and following surgical procedures can reduce treatment complications and improve outcomes; insulin use often plays a key role in achieving this objective [13, 14]. Several ‘high-risk factors’ for insulin errors along the peri-operative journey have been identified and described in national reports [7, 8, 15]. Patients admitted under surgical specialties have been identified as having a higher risk of developing hospital-acquired Diabetic-Ketoacidosis, associated with mismanagement of insulin during admission [12]. Nationally, there is a focus on improving perioperative care for PWD [14].

#### Older age, frailty and diabetes

Data from NADIA highlights over two thirds of PWD admitted to hospital were 65 years of age and above [16]. In the *Highs and Lows* report reviewing surgical care, patients median age was 69 years; two fifths were identified as vulnerable or frail [15]. Older or frail adults with diabetes are at greater risk and more vulnerable in the context of insulin errors. Diabetes in older age is frequently associated with other features such as co-morbidities, frailty, polypharmacy, functional deficits in relation to: cognitive and physical function, and communication (hearing loss and vision) [17].

Frailty is characterised by the reduction of physiological reserve and resilience to recover from physical and or psychological stressors such as illness or injury. This in turn leads to a state of increased risk [17, 18]. Frailty has been associated with greater risk of post-surgical complications and increased LOS. Frailty and other age related deficits can increase risks of hyper or hypoglycaemia [19]. There may also be reduced diabetes self-management capacity leading to the need for third-party administration of insulin therapy in hospital, increasing risks for error. Therefore, in this study the aim will be to develop a multimodal intervention to reduce hospital insulin errors in older PWD in perioperative care settings.

#### Co-designing a model to develop a multimodal intervention to reduce hospital insulin errors

Traditional approaches to incident reviews tend to be based on developing a linear explanation to the sequence of events precipitating the error. However, in complex adaptive systems such as hospital surgical settings, multiple interacting components need to be considered to enhance system resilience [9]. Compassionate engagement and involvement of those affected by incidents is central to the NHS’s new Patient Safety Incident Response Framework, as is an exploration of events to understand and strengthen systems [20]. In this study we will co-design a model for a multimodal intervention addressing this underpinning complexity by considering patient, HCP and systemic factors that contribute to insulin errors in the context of older people in perioperative care settings.

SHINE study will use a co-design approach using design thinking principles [21–25]; this approach aims to develop creative ideas and solutions by collaboratively involving key stakeholders and end users in the design process including decision-making. Design thinking has its origins in architecture and industrial design, with more recent application to healthcare [21, 26, 27]. One way of conceptualising design thinking is to consider 3 spaces or phases of innovation process: Inspiration, Ideation and Implementation (Table 1 outlines characteristics of these) [24, 25].

**Table 1:** Characteristics of design thinking phases [24, 25].

Phases of Design Thinking	Characteristics	Core Principles
**Inspiration** *(This study)*	Engagement with key stakeholders Empathise: fostering a culture of care and understanding/sharing in the experiences of patients, carers and staff Framing design challenge Learning and exploration Relationship building	Empathy, Optimism, Iteration, Creative Confidence, Making, Embracing Ambiguity, and Learning from Failure
**Ideation** *(This study)*	Share learning from Inspiration phase Sense making Identifying opportunities for design Generation of ideas; some to keep some to discard Prototype development Multiple iteration and refinement cycles (divergent and convergent thinking)	
**Implementation** *(Future study)*	Testing prototype developed in Ideation phase Partnerships Monitoring, feedback and evaluation	

Characteristics of Design Thinking Phases: Inspiration (this study), Ideation(this study), Implementation (future study) [24, 25].

**Fig 1:**
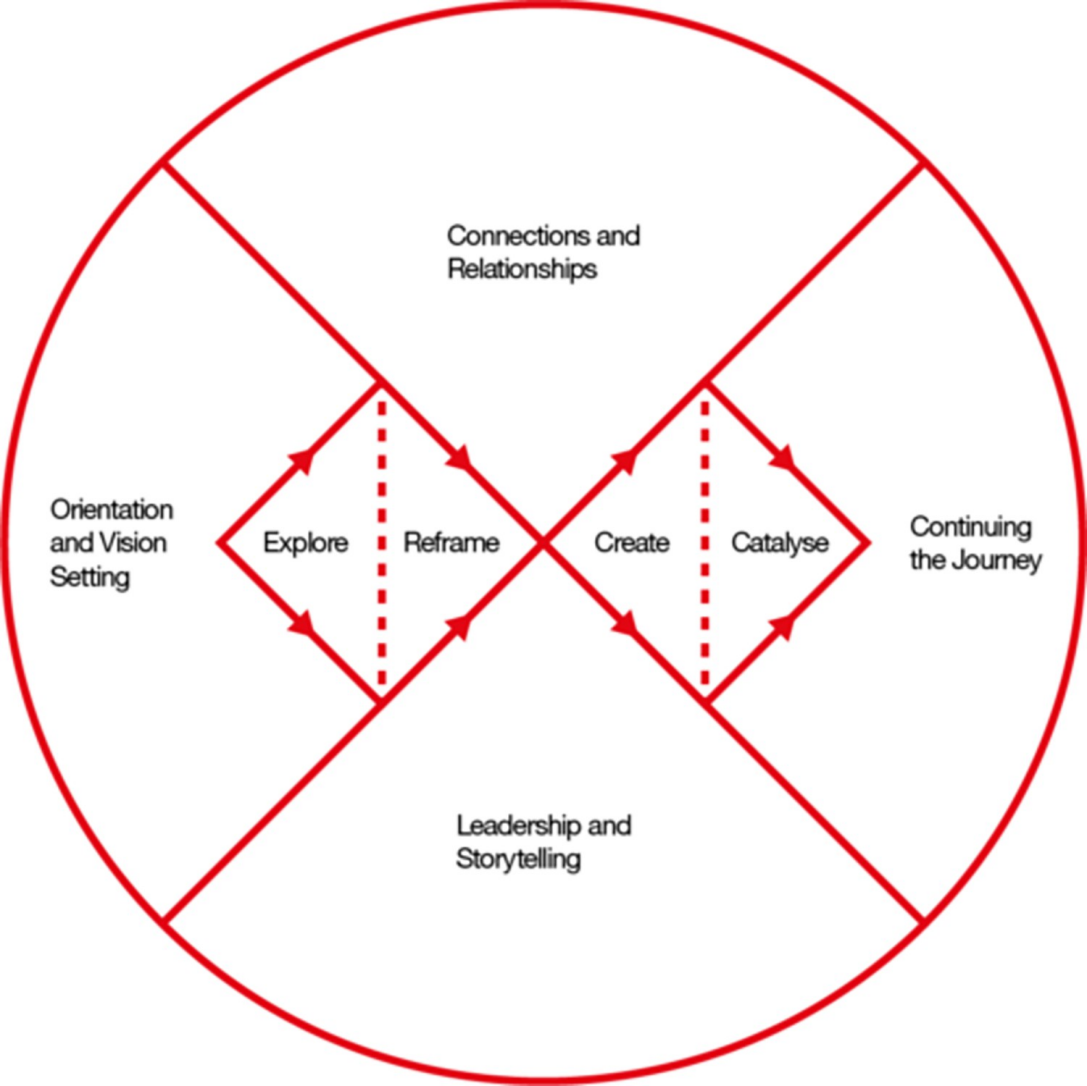
Design council systemic design framework. British Design Council Systemic Design Framework. This work by the Design Council is licensed under a CC BY 4.0 license [23].

The British Design Council’s [23] Systemic Design Framework, represented in Fig 1, is a helpful guide to design thinking principles for the non-designer. Building on from the double diamond divergent and convergent design thinking process, it also considers non-linear dynamics in complex systems. Design thinking process can involve a range of data collection methods including observations, interviews and workshops [21, 22, 26].

### The study aims and objectives

The overall aim of this study is to co-design with PWD, HCPs and other stakeholders (family members/carers) a conceptual model for a multimodal intervention to reduce insulin errors in older PWD treated with insulin undergoing a surgical hospital admission.

The study objectives are to:

- Elicit the experiences and perspectives of older or frail adults with diabetes and HCPs in relation to insulin use in surgical care settings.
- Identify key factors involved in insulin safety and errors, from the perspectives of patients and HCPs.
- Model factors that contribute to insulin errors in the context of perioperative care for older people using insulin.
- Elicit potential solutions and intervention components from patients, HCPs and other stakeholders.
- Develop an intervention framework modelling potential intervention components to the factors associated with insulin errors.

## Methods

### Study and protocol registration

This study has been registered on Open Science Framework: https://osf.io/4wvu5.

### Study design

**Fig 2:**
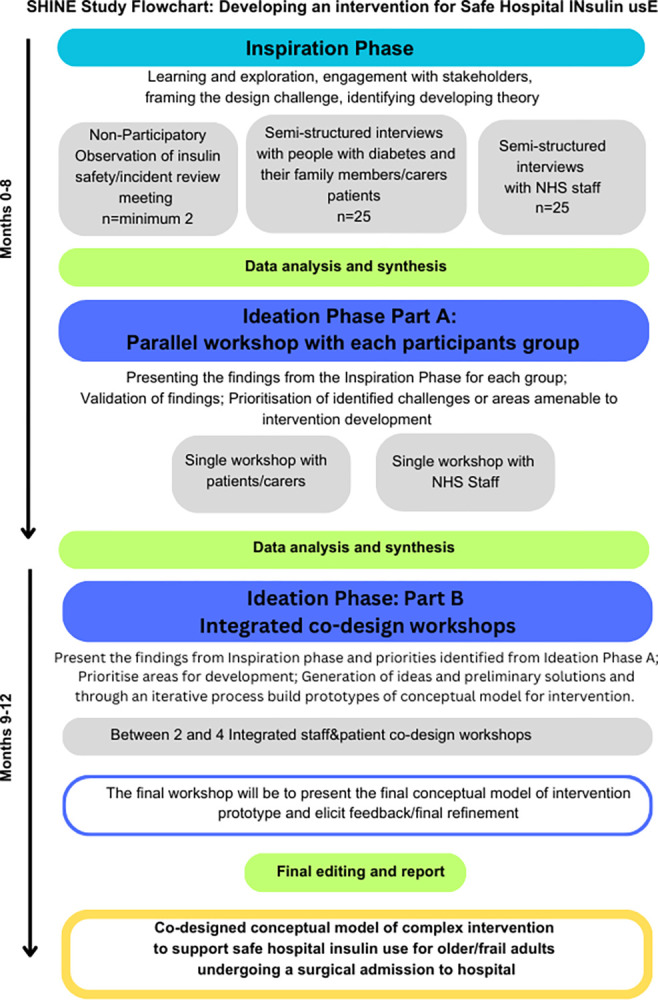
SHINE study flowchart. SHINE Study Flowchart: Developing an intervention for Safe Hospital Insulin Use.

The study design has been informed by the National Institute for Health Research (NIHR)/UK Medical Research Council (MRC) Framework for development and evaluation of complex interventions [28]. This qualitative study focuses on the theory and modelling phases of the framework. A co-design approach, using design thinking process principles, will be used to build an intervention model. SHINE study is comprised of 2 phases following the Inspiration and Ideation phases of the design thinking model (see study flowchart in Fig 2). Implementation phase would be addressed in a future study.

Phase 1 Inspiration: the focus is on learning, exploration and engagement with stakeholders, to model the factors and processes that contribute to insulin errors. A multi-method approach will be used, incorporating non-participant observations of incident review meetings and semi-structured interviews with PWD, HCPs and other stakeholders. Modelling will also be informed by the findings of our scoping review.

Phase 2 Ideation: Findings from the Inspiration Phase will be used to generate ideas for the intervention components targeting the different factors identified. Ideation phase is divided into two substages: A and B. Substage A: will involve parallel workshops with patients and HCPs; in substage B, integrated workshops will bring patients and HCPs together to refine and prioritise the intervention components. The different workshops and participants for this phase of the study are presented in Table 2.

**Table 2:** Types of workshops to be held in SHINE study.

Phase	Type of workshop	Who will attend	Aim of workshop
**Ideation Phase:** **Substage A:** Parallel workshop with each participants group	Single workshop with patients/their carers Duration of workshop: lasting up to 2h	Patients who were interviewed will be invited to attend this workshop.	• Presenting the findings from the Inspiration *Phase amongst patients* • Validation of findings• Prioritisation of identified challenges or areas amenable to intervention development• Establishment of relationships and connections for future co-design workshops
	Single workshop with NHS staff Duration of workshop: lasting up to 2h	• NHS staff who were interviewed will be invited to attend this workshop.	• Presenting the findings from the *Inspiration Phase amongst NHS staff* • Validation of findings• Prioritisation of identified challenges or areas amenable to intervention development• Establishment of relationships and connections for future co-design workshops
**Ideation Phase:** **Substage B:** Integrated co-design workshops	Between 2 and 4 Integrated patient-staff co-design workshops Duration of workshop: lasting up to 2h	• Patients who were interviewed will be invited to attend. • NHS staff who were interviewed will be asked for expression of interest to attend these workshops • Patients and NHS staff will be active participants in these workshops and may also be working alongside each other in smaller groups within the workshop.	• Present the findings from Inspiration phase • Present the priorities from the parallel workshops with each participant group *(ideation phase substage A)* • Prioritise areas for development• Generation of ideas and preliminary solutions and through an iterative process build prototypes of conceptual model for intervention• The final workshop will be to present the final conceptual model of intervention prototype and elicit feedback/final refinement prior to being ready to proceed to testing phase in a future study

Types of workshops held at the different phases of SHINE Study; participants and aims of workshops

### Research approach

This study draws from the NIHR/MRC Framework for development of complex interventions [28]. Methodologically, it employs Participatory Research utilising a co-design approach based on design thinking principles [22–24] and drawing upon Experience Based Co-Design process [27]. Given the highly individualised nature of insulin treatment; self-management, along with caregiver support, plays a crucial role. Insulin errors in hospital affect inpatient experience. Thus, it becomes crucial to involve patients/carers and staff in decision-making in the intervention development; listen to their narratives, experiences, insights and co-design the intervention together, aspiring to produce better experiences of care. Thus, a ‘partnership’ approach to intervention development using co-design appears appropriate for this study [27, 28].

### Study setting and recruitment

The study will be conducted in the surgical wards (including general surgery and frailty trauma and orthopaedic wards) of a NHS district general hospital in England. The hospital is situated in a rural county with a higher prevalence of older adults with diabetes compared to other hospitals in England. Thus far, most insulin safety interventions have been developed in large or teaching hospitals in more urban areas (unpublished data). Recruitment strategy was planned with guidance from the lead diabetes consultant (LDC) at the research site and is presented below for each of the study phases. Recruitment began 12 April 2024 and is ongoing.

### Study sample and inclusion and exclusion criteria

Study participants will be up to 25 older people with diabetes with recent experience of inpatient surgical care and their family members/carers and up to 25 HCPs.

Study inclusion and exclusion criteria are outlined in Table 3.

**Table 3:** Inclusion and exclusion criteria in SHINE study.

Potential Participants	Inclusion Criteria	Exclusion Criteria
**Service user/Patient inclusion criteria**	●Are able to give informed consent ●Age ≥ 65 years OR frailty identified/documented on admission ●Patient with a diagnosis of diabetes before their hospital admission and treated with insulin therapy before and during hospital stay ●History of hospital admission for major orthopaedic and general surgical procedures requiring minimum 1 night hospital stay post-surgery at study site selected surgical wards: within the last 9 months. ●Fluent English speaker. ●Portuguese speaking patients can be included at interview stage	●Unable to give informed consent ●Patients that don’t have a formal diagnosis of diabetes prior to their hospital admission ●Patients with diabetes not treated with insulin therapy prior to and during their hospital stay ●Under age 65 years if absence of frailty on admission ●Unable to communicate effectively in English/Portuguese for interview stage ●Unable to communicate effectively and fluently in English for the workshops and co-design events ●Don’t have history of hospital admission for major orthopaedic and general surgical procedures requiring surgery with minimum 1 night hospital stay post-surgery within last 9 months at study site designated wards ●Patients undergoing day surgery without an overnight stay and patients who had a minor procedure will be excluded. ●History of hospital admission at the study sites but not for major orthopaedic and general surgical procedures and not admitted to the study site designated wards ●Patients who do not meet inclusion criteria
**Family member/carer inclusion criteria**	●Family members such as older or frail patients’ children, siblings, spouses, friends or carers who speak and understood English and are involved in the usual diabetes care/insulin management of eligible patients may be considered for inclusion if invited by the patient/service user	●Patient does not invite/consent to family members or carers being involved ●Family member/Carer unwilling to participate ●Family member/Carer who do not meet inclusion criteria
**NHS Staff inclusion criteria**	●Clinical and non-clinical staff involved in the hospital care/transfer of care/insulin incident or safety review of older or frail adults with diabetes undergoing surgical admission at the study site designated wards ●Working full or part time at the study sites (for a minimum of 3 months)	●Clinical and non-clinical staff not involved in the hospital care/transfer of care/insulin incident or safety review of older or frail adults with diabetes undergoing surgical admission at the study site designated wards ●Employed at the study sites for less than 3 months ●Locum and agency staff ●For the non-participatory observations of incident review/safety meetings: staff who opt out ●NHS staff who do not meet inclusion criteria

Participants will be sampled purposively to include a range of surgical procedures and experiences [29, 30]. HCP participants will be recruited from a range of different grades, professional roles, experience of insulin use in surgical settings; and those involved in safety reviews of insulin errors occurring in older or frail adults with diabetes undergoing surgical admission. Purposive sampling of patients with diabetes/their carers who have had a surgical admission at the study site will recruit participants with different types of diabetes and insulin treatment modalities; varying levels of frailty, duration of hospital admission, and experience of insulin errors. Frailty will be identified and classified using the Rockwood clinical frailty score. This 9-point scale, was developed to summarise the level of fitness or frailty of an older adult, following evaluation by a healthcare professional [18]. This scale is embedded in routine clinical practice at the research site.

Carers/family members are welcome to take part in this research, however their participation will be determined by the individual patients/service users who choose to take part and if they wish to invite a carer. The term carer will be defined by the patient i.e. whoever they choose to bring as their carer.

### Ethical considerations

#### Ethics

Ethical approval was obtained prior to commencement of study. Application for King’s College London to act as sole study sponsor was gained. NHS Health Research Authority ethical approval from East Midlands-Derby Research Ethics Committee (24/EM/0022) was gained.

A one off £15 Amazon/Love to Shop voucher will be provided to PWD as a thank-you for participation in the study and to HCP participating out of their usual working hours. Light refreshments will be provided at co-design events. Patients attending research activities will be reimbursed for reasonable travel expenses.

#### Dissemination

Varied strategies to disseminate study findings to relevant stakeholders including: patients, patient organisations; local and national NHS networks; and diabetes clinical research networks will be employed, including:

- Patient and professional summaries; study participants will be able to access a final study report if they wish;
- Findings will be shared with patient organisations such as Diabetes UK, Healthwatch;
- Clinical research and professional channels–national/international conferences, network meetings; peer-reviewed professional/academic journals;
- Final PhD thesis report;

#### Patient and public involvement

Patient involvement has been sought at various stages through development of this study. Informal conversations with patients and staff, discussion at a local peer support group for people living with Type 1 Diabetes; and Healthwatch Herefordshire have gauged and confirmed acceptability of the research, informing and shaping its design. Study documentation was developed with patient input.

By using a co-design approach, patients and staff involved in the feedback/co-design workshops will have a ‘voice’ in selecting key priorities/areas the intervention to develop will address.

## SHINE study phases: Relevant data collection and analysis

### Phase 1: Inspiration

Phase 1 includes non-participant observations and interviews with patients and staff (Fig 2); these phases will be further described below.

### Non-participant observations

The purpose of the observations will be framed as an opportunity to learn more about the insulin incident and safety review process ahead of interviews and workshop events, so that hospital insulin safety context is better understood. Observations will focus on type, amount and quality of activities, interactions and organizational processes. Notwithstanding organizational and operational capacity to accommodate observations, attempts will be made to observe a minimum of 2 meetings taking place at the beginning of Inspiration phase.

#### Sample

Staff in attendance during the meetings observed.

#### Recruitment and informed consent

For the observations, the lead researcher (CLF) will liaise with the LDC, Medicines Safety Officer and Matrons/Ward Sisters regarding attendance of relevant meetings.

Observations will be on an opt-out basis, i.e. if a person is unhappy about being part of the observations, they can alert the meeting chair/researcher, and observations will be terminated. No individual written consent will be obtained for collection of observational data, given observational data collected does not involve direct patient contact or information about identifiable staff or patients. The researcher/meeting chair will ask staff before each observation whether anybody has chosen to opt out.

Posters giving information about the study and observations will be circulated and displayed in communal staffing areas, with information about where they can access further information should they wish.

#### Data collection

Contemporaneous or post observation field notes will be collected by CLF guided by an observation tool. CLF will be a non-participant observer to minimise interference of clinical care discussions/interactions and facilitate ease in recording field notes.

An adapted version of the AEIOU framework [22] will be used to guide observations (Table 4). This will allow to draw from relevant core theory into a framework which allows structured observation and recording of data. AEIOU Framework is an adaptable tool used in design thinking, particularly in the early inspiration phases, to provide structure and guidance to observational fieldwork [22, 31].

**Table 4:** AEIOU framework and areas to be observed.

AEIOU Framework: Areas to be observed	Description
**Activities**	Will take into account the various actions and tasks that take place during the meetings, from what happens in the meeting, what activities and tasks are undertaken to how the insulin related incidents are presented, described, and explored, to what actions and decisions are taken following the review.
**Environment**	Considers the characteristics and functionality of the space/environment the meetings and safety review takes place in.
**Interactions**	Relates to interactions between users, system(s), interfaces.
**Objects**	Relates to the tools, equipment and resources used.
**Users**	Relates to the key & relevant stakeholders, their roles, influencing factors for them in the context of insulin safety review.

AEIOU Framework and areas to be observed [22] A: Activities, E: Environment; I: Interactions; O: Objects; U:Users

#### Data analysis

Field note observations will be digitally transcribed as soon as possible by CLF. NVIVO software will be used to aid management of data during analysis.

Data will be coded and analysed to create a coding matrix to consider areas such as general details of incidents, observations around policies and processes, interactions between users, learning outputs from the review and any actions that resulted from the review. If, during coding, additional categories for the matrix are identified, these will be added iteratively.

### Interviews with patients and staff

Semi-structured interviews will aid the researcher to gain greater understanding into participants’ subjective experiences and perspectives. Findings from the interview phase will be used as a basis for future phases of the co-design work.

#### Sample

The purposive sample will aim to include:

- Up to 25 people with diabetes and their family members/carer
- Up to 25 members of NHS staff.

Upon reflection, and in order to ensure more equitable distribution of influence and experiences, we made the decision to increase the number of individuals with diabetes to align with the number of participating HCPs. By doing so, we aimed to enhance the overall value of the study and foster a balanced dynamic that maximises the contributions of all involved stakeholders.

Malterud’s *Information power* (IP) Model [32] was used to guide sample size and will be used to evaluate sample size during the interview stage [32, 33].

The concept of IP alludes to the more information pertinent to the study held within the sample, the lower the sample size needs to be, and vice versa [32]. Five dimensions impact on IP: the *study aim*, the *sample specificity* the *use of established theory*, *quality of dialogue* and *analysis strategy* [32].

The defined aims of this study, purposive sampling with defined inclusion/exclusion criteria, a-priori theoretical background informing the study, use of interview topic guides and in-depth qualitative analysis will help give IP to the sample, looking to achieve a robust set of perspectives on the problem being explored.

#### Recruitment and informed consent

We have developed a recruitment strategy for the study in consultation with the LDC at the research site. This strategy is outlined below:

### Patient Recruitment

1. During routine clinical care amongst eligible patients who have undergone a surgical admission to the study designated wards, relevant surgical and diabetes team clinicians will inform patient of the study.
  OR
2. Eligible patients identified through retrospective review of inpatient database/log of activities will be contacted by a clinical member of the diabetes team to mention the study to the patient. Activities relevant to this may include review of surgical lists, point of care glucose testing data, diabetes safety incidents or diabetes inpatient data activities.
  Using their professional judgment, the clinician will seek verbal agreement from patient to introduce CLF to them. If they are interested, CLF will see/contact patient, introduce the study, provide participant information sheet (PIS) and consent form (CF).
  OR
3. Self-referral using contact details on recruitment posters/information advertised. CLF will see/contact patient, introduce study, provide PIS and CF.
  Patients will be given sufficient time to make a decision, after which a written informed consent will be obtained if they decided to participate.

### Carer Recruitment

Patients included in the study can invite 1 carer/family member to research activities. If they are interested, they will be provided with PIS and CF.

Carers will be given sufficient time to make a decision, after which a written informed consent will be obtained if they decided to participate.

### NHS Staff Recruitment

1. The study will be promoted via recruitment posters at the hospital site via official communication methods (email, newsletter, trust’s social media, physical posters).
  OR
2. Clinicians will also be recruited via team leaders, local diabetes team, word of mouth, email communications promoting project.
  OR
3. Self-referral.
  Once self-referral or verbal consent to contact has been established, CLF will make contact, provide potential participants with a brief overview of study, and answer any questions. They will be given a PIS and CF and given sufficient time to make a decision, after which a written informed consent will be obtained if they decided to participate.

#### Data collection

Data will be collected and analysed by CLF and study team. To describe the study sample and contextualize contributions, patient and staff participant demographic profile will be collected. Participants’ identity will be anonymised by allocating study identifier codes.

Semi-structured, audio-recorded interviews with patients and staff, lasting up to an hour can be held face to face or virtually depending on participant preference. These will allow the researcher to set topics/themes to be explored in the interaction but allowing for flexibility in the exploration, based on participant’s responses.

The observations and relevant literature review will help inform development of an interview topic guide. Use of probing questions will be employed to explore topics in depth.

#### Data analysis

Audio-recordings will be transcribed; data will be analysed using Framework Analysis (FA), a widely used analytical approach involving the systematic organizing of data within a pre-set framework of themes [34]. The Richie and Spencer 5-Step process, summarised in Table 5 will be used: Familiarization, identifying of a thematic framework, Indexing/Coding, charting, mapping and interpretation [34]. FA has been used in other co-design research [29].

**Table 5:** Summary of steps for thematic analysis using Framework Analysis process (Ritchie and Spencer, 1994).

Thematic analysis using Framework Analysis process (Ritchie and Spencer, 1994)	
**Step 1:** **Familiarization**	Full immersion in the data. Active reading/listening and re-reading/ /listening of the data (transcriptions, notes) to become familiar with depth and breadth of the data, taking initial notes, ideas on the data. Key ideas and recurrent themes are listed.
**Step 2:** **Identifying a thematic framework**	Devising and development of the thematic framework (also referred to as index) is an iterative process. Drawing from apriori theoretical constructs; themes which have emerged from the literature review and the familiarization process: key issues, concepts and themes will be identified to which the data can be examined/referenced. This will help sift and sort the data within a thematic framework.
**Step 3:** **Indexing/Coding**	This phase relates to systematic application of the thematic framework to the data in textual form/transcripts. Data is read and indexed according to the thematic framework. The framework will be systematically and continually revised until the data from all the transcripts is captured. A codebook of themes will be developed and modified by the researcher following an iterative process.
**Step 4:** **Charting**	Once the transcripts have been indexed a picture of the data as a whole is constructed. Charts with headings and subheadings are constructed. In the thematic approach charts are constructed for each key subject/theme area. The data will be entered into framework matrices for each theme in NVivo.
**Step 5:** **Mapping and interpretation**	During this phase, the key dimensions within the data will be identified, pulling together key characteristics and mapping, analysing and interpreting the data as a whole.

Summary of steps for thematic analysis using Framework Analysis process [34]

NVIVO software will be used to aid management of data during analysis.

This study will also draw from Complex Systems thinking, Safety 2 and Resilient Healthcare principles in developing the intervention and in the theory based analysis [9]. Using safety 2 and resilient healthcare principles to learn from incidents has been found to focus attention on ways of strengthening systems prospectively [35]. Further theoretical lenses may be used, guided by data and priorities emerging from the research process.

Findings will be synthesised and combined ready for presentation to participants in phase 2 of the research.

## Engagement activities between inspiration and ideation phases

Between inspiration and ideation phases, engagement activities amongst participants will take place to continue keeping momentum and potentially consult/discuss early thoughts from interviews/workshops in preparation for workshop events. Amongst these, telephone calls, email, online whiteboard or other means appropriate and accessible to participants may be used.

## Phase 2: Ideation

Ideation phase is subdivided in 2 parts: Substage A (parallel workshops with patients and staff) and B (patient-staff-integrated co-design workshops) (Table 2).

This phase of the study uses findings from Inspiration phase as a springboard for the co-design groups to generate ideas, possible solutions and prototypes [21, 22, 26]. The prototype or conceptual model of an intervention to support hospital system-based insulin safety/incident exploration, learning and response will be produced at the end of this phase.

### Substage A: Single parallel workshops with patients and staff

In this phase, findings from the *Inspiration Phase* amongst patients and staff will be presented individually to each group. This will enable validation of findings and prioritisation of identified challenges or areas amenable to intervention development.

### Sample

- Up to 25 patients for the workshop with patients and their family member/carer
- Up to 25 members of NHS Staff for the workshop with staff

It is anticipated the numbers of participants in workshop events will be smaller than in interview phase, as some study drop-out anticipated.

#### Recruitment and informed consent

Recruitment strategy for the study has already been presented above in Phase 1. If required, further recruitment will be instigated during Phase 2.

#### Data collection

Both workshops will be informed by published tool-kits to support co-design process [22]. There will be a commitment to giving everyone a voice and take all voices seriously.

CLF will be present at all events, carry out facilitation and moderation of the workshops, supported by a member/s of the diabetes team who will provide general support with note taking, planning and management of events. Main presentations at events will be audio-recorded. Materials and ‘presentations’ created through activities used in the workshops will be included as data to be analysed.

#### Data analysis

Audio recordings will be transcribed. NVIVO software will be used to aid management of data during analysis. Qualitative data will be analysed using FA process summarised in Table 5 [34].

Findings will be synthesised and combined ready for presentation to participants in substage B of this phase.

### Substage B: Patient-staff-integrated co-design workshops

Between 2 and 4 workshops will be held (Table 2), depending on the iterative intervention refinement process. Findings from Inspiration phase and priorities from the parallel workshops with each participant group (ideation phase substage A) will be presented. Areas for development will be prioritised. There will be generation of ideas and preliminary solutions and through an iterative process prototypes of conceptual model for intervention will be developed.

The final workshop will be to present the final conceptual model of intervention prototype and elicit feedback/final refinement prior to being ready to proceed to testing phase in a future study.

#### Sample

- Minimum 6–8 patients and NHS staff participants.
- Patients can invite 1 family member/carer to research activities.

Co-design groups/workshops usually have smaller numbers of participants, hence the minimum target sample for each workshop [27].

#### Recruitment and informed consent

For the workshops, all patients who were interviewed will be invited. NHS staff who are interviewed will be invited to attend the staff workshop; they will be asked to express if they have an interest in being involved in the integrated staff-patient co-design workshops/process. Depending on interest and number of patients agreeing to participate in the co-design workshops, a sample of staff as representative as possible of different roles and experiences will be selected. Efforts will be taken to have, as much as is feasible, a similar number of patients/carers and staff and variety of staff roles represented in the integrated patient and staff co-design workshops, noting the risk of power imbalances and of perspectives represented.

Numbers of participants in co-design studies vary depending on scale and number of sites but total numbers in this study are similar to other co-design studies when considering it is a single site study [26, 30].

#### Data collection

Workshops will be informed by published tool-kits to support the co-design process [22]. Activities such as empathy maps, brainstorming, journey map, storyboard etc are likely to employed. There will be a commitment to giving everyone a voice and take all voices seriously.

CLF will be present in all events, carry out facilitation and moderation of workshops, supported by a member/s of the diabetes team who will provide general support with note taking, planning and management of the events. Main presentations at events will be audio-recorded. Materials and ‘presentations’ created through activities used in the workshops will also be included as data to be analysed.

#### Data analysis

Audio recordings will be transcribed. NVIVO software will be used to aid management of data during analysis. Qualitative data will be analysed using FA process summarised in Table 5 [34].

Over the course of the co-design workshops and subsequent summarising and synthesis of data, a logic model of the intervention will be built iteratively in a collaborative process between study participants and study team. This will be presented in the final co-design workshop for validation and final refinement.

## Discussion

Insulin errors in hospital still occur frequently for inpatients with diabetes, putting them at risk of severe harm and negatively impacting their inpatient experience [8, 11, 12]. Evidence has shown recurrent errors persist at multiple stages of the peri-operative journey [7, 8, 15, 36]. Complexity around hospital insulin use has been presented, highlighting risks with older or frail adults undergoing a surgical admission; continual concerted multi-pronged efforts to improve safety are required. The need for insulin safety interventions which increase system resilience and for greater patient voice/participation in design of insulin safety interventions have been identified [37, 38].

Further work on developing system-based learning and response to insulin safety incidents could support prospective system-based resilience and improve insulin safety. Developing a multi-modal intervention to support system-based exploration of hospital insulin use and errors, arguably enables a better understanding of system use of insulin, and may identify touchpoints and patterns of interactions where system resilience could be enhanced, thereby improving insulin safety and inpatient experience.

Ensuring patients/carers and staff are involved in the process of developing the intervention through a co-design approach gives active voice in the choice of priorities and intervention components to develop. Observational fieldwork will help build and understand context ahead of other research activities. Furthermore, observations establish what people say and do in practice, rather than what they say they do; whilst acknowledging that as participants are aware they are being observed, behaviours may change. Exploratory work with HCP through interviews and workshops will enable a deeper understanding of how HCPs engage with and view the insulin use process and insulin review/learning from incidents. In depth interviews and workshop with patients/carers will ensure their voice and priorities are understood and captured. Their ongoing participation in the co-design workshops ensures the intervention does not lose sight of the principal people the intervention seeks to support.

Whilst this study is only taking place in one hospital site, there is an acknowledgement that many insulin safety related issues are common to various hospitals. The proposed study would present a novel contribution towards hospital insulin safety.

## Conclusion

This study will contribute novel insights regarding insulin safety by exploring and developing further understanding of the experiences and priorities for safe peri-operative hospital insulin use for older or frail PWD and NHS staff looking after them. By co-designing the intervention, relevant stakeholders such as older or frail adults with diabetes, their carers and NHS staff will have a voice in shaping priorities and intervention components to be developed.

This study will produce a novel intervention model for a complex intervention to support the insulin safety review process, to improve the experiences and safety of PWD treated with insulin undergoing a surgical hospital admission and reduce insulin errors, increasing insulin use system resilience and safety.

The findings of this co-design study will provide an intervention template we can take forward to a feasibility study to help improve hospital insulin safety and experience for PWD and support staff looking after them.

## Supporting information

S1 File Ethics approved protocol SHINE study. (PDF)
