# Supplementary material for: SHINE study: Developing an intervention for safe hospital insulin use for older or frail adults with diabetes undergoing surgical hospital admission: Study protocol
Source: PLoS One. 2024 Dec 12;19(12):e0315387. doi: 10.1371/journal.pone.0315387 (PMC11637288; doi:10.1371/journal.pone.0315387)
Supplement: S1 File — (PDF) [file pone.0315387.s001.pdf]

SHINE Study: Developing an intervention for **s**afe **h**ospital **in**sulin us**e** for older/frail adults with diabetes undergoing a surgical admission to hospital.

This protocol has regard for the HRA guidance

FULL/LONG TITLE OF THE STUDY

SHINE Study: Developing an intervention for **s**afe **h**ospital **in**sulin us**e** for older/frail adults with diabetes undergoing a surgical admission to hospital.

SHORT STUDY TITLE / ACRONYM

SHINE Study: Developing an intervention for **S**afe **H**ospital **IN**sulin us**E**

PROTOCOL NUMBER AND DATE

Version 1.0 12 December 2023

RESEARCH REFERENCE NUMBERS

**IRAS Number:**	334058

SIGNATURE PAGE

The undersigned confirm that the following protocol has been agreed and accepted and that the Chief Investigator agrees to conduct the study in compliance with the approved protocol and will adhere to the principles outlined in the Declaration of Helsinki, the Sponsor's SOPs, and other regulatory requirement.

I agree to ensure that the confidential information contained in this document will not be used for any other purpose other than the evaluation or conduct of the investigation without the prior written consent of the Sponsor.

I also confirm that I will make the findings of the study publicly available through publication or other dissemination tools without any unnecessary delay and that an honest accurate and transparent account of the study will be given; and that any discrepancies from the study as planned in this protocol will be explained.

Chief Investigator:

Signature: .... Date: ....../....../......

Name: (please print):

....

LIST of CONTENTS

GENERAL INFORMATION	Page No.
TITLE PAGE	2
RESEARCH REFERENCE NUMBERS	2
SIGNATURE PAGE	3
LIST OF CONTENTS	4
KEY STUDY CONTACTS	5
LIST OF ABBREVIATIONS AND DEFINITIONS	6
STUDY SUMMARY	7
FUNDING	10
ROLE OF SPONSOR AND FUNDER	11
ROLES & RESPONSIBILITIES OF STUDY STEERING GROUPS AND INDIVIDUALS	12
STUDY FLOW CHART	13
STUDY GANTT CHART	15
SECTION	
1. BACKGROUND	16
2. RATIONALE	19
3. THEORETICAL FRAMEWORK	20
4. RESEARCH QUESTION/AIM(S)	23
5. STUDY DESIGN/METHODS	24
6. STUDY SETTING	29
7. SAMPLE AND RECRUITMENT	30
8. ETHICAL AND REGULATORY COMPLIANCE	35
9. DISSEMINATION POLICY	42
10. REFERENCES	43
11. APPENDICES	48

KEY STUDY CONTACTS

Chief Investigator	Dr Kirsty Winkley<br>Care in Long Term Conditions, Faculty of Nursing, Midwifery & Palliative Care, King's College London, James Clerk Maxwell Building; Waterloo Road; London; SE1 8WA, UK<br>Email: kirsty.winkley@kcl.ac.uk
Lead Researcher	Mrs Christina Lange Ferreira<br>Care in Long Term Conditions, Faculty of Nursing, Midwifery & Palliative Care, King's College London, James Clerk Maxwell Building; Waterloo Road; London; SE1 8WA, UK<br>Email: christina.lange_ferreira@kcl.ac.uk<br>Phone: 07572965320
Sponsor	King's College London<br>Professor Bashir Al-Hashimi<br>Room 8.11, 8th Floor Melbourne House<br>44-46 Aldwych<br>London WC2B 4LL<br>Telephone: +44 (0)207 848 7306<br>Email: vpri@kcl.ac.uk
Funder(s)	**PhD fees and Salary (funding secured):**<br>Foundation of European Nurses in Diabetes<br>Registered Charity no. 1134995<br>Company limited by guarantee<br>Registered in England no. 7114723<br>Registered Office: 37 Earls Drive, Newcastle on Tyne, NE157AL, UK<br>Tel: +44 (0)2088766122<br>**Study Participant thank-you vouchers (funding secured):**<br>Herefordshire and Mid-Powis Diabetic Care Fund<br>Registered Charity no: 516561<br>42 Rural Enterprise Centre<br>Vincent Carey Road<br>Rotherwas<br>Hereford<br>HR4 0LQ<br>Phone: 01432345703
Key Protocol Contributors	Mrs Christina Lange Ferreira<br>Dr Kirsty Winkley<br>Professor Angus Forbes<br>Dr. Hellena Habte-Asres<br>Dr Sara Donetto

LIST OF ABBREVIATIONS AND DEFINITIONS

A&E Accident & Emergency

AGM Annual General Meeting

CI Chief Investigator

COVID-19 Coronavirus (COVID-19) pandemic

CQC Care Quality Comission

DKA Diabetic KetoAcidosis

EBCD Experience Based Co-Design

FEND Foundation of European Nurses in Diabetes

GDPR General Data Protection Regulation

GIRFT Getting it right first time

HCP Healthcare Professionals

HHS Hyperosmolar Hyperglycaemic State

HRA Health Research Authority

ISMP Institute for Safe Medication Practices

ITU Intensive Therapy Unit

IV Intravenous

KCL King's College London

MRC Medical Research Council

NADIA National Diabetes Inpatient Audit

NCEPOD National Confidential Enquiry into Patient Outcome and Death

NHS National Health Service

NIHR National Institute for Health Research

PhD Doctor of Philosophy

PI Principal Investigator

PIS Participant Information Sheet

PPI Patient and Public Involvement

PSIRF Patient Safety Incident Response Framework

R&D Research and Development

REC Research Ethics Committee

RESILIENT framework InteRacting componEntS in InsuLIn usE In hospiTal framework

SHINE Study: Developing an intervention for **S**afe **H**ospital **IN**sulin us**E**

UK United Kingdom

WVT Wye Valley NHS Trust

STUDY SUMMARY

Plain English Summary

Background

Diabetes is a long-term health condition affecting how the body regulates glucose. Insulin, a hormone produced by the pancreas, enables the body to use glucose and convert this into energy. Insulin injections, usually self-administered, are an important part of treatment for many people with diabetes. Having good blood glucose levels before, during and after surgery, can reduce chances of infection, complications and longer hospital stay.

Insulin is considered a high-risk medication due to the high level of harm that can occur in case of an error (for example dangerously low or high blood glucose levels).

Unfortunately, insulin related errors in hospital are common. Nationally, 2 in 5 inpatients treated with insulin experience an error. Many healthcare professionals feel they do not know enough about insulin. People with diabetes can feel anxious about their insulin treatment in hospital. Older/frail adults have potential characteristics which increase the risk for insulin error.

Aims

This study will contribute towards the development phase of an intervention to improve insulin safety during a surgical admission to hospital. The population of interest are older/frail people with diabetes (and their family member/carer) with experience of a surgical admission and NHS staff looking after them.

This study aims to:

- Explore and understand the needs of patients and staff regarding hospital insulin safety and learning from insulin related incidents and how to improve patient experiences of hospital insulin safety
- Identify how to best support the insulin safety review process in hospital to strengthen hospital insulin safety and prevent insulin errors

Methods

To carry out this qualitative study, a co-design approach will be used, bringing staff and patients (and their carers) together in the process. A range of data collection methods such as observations, semi-structured interviews and co-design workshops with staff and with patients. The study will take place at a single NHS hospital in the West Midlands. Patients and public (PPI) views and preferences have been sought. These have been incorporated into the design of the study.

Results

This study is part of a doctoral research programme and will be reported in the final PhD thesis. In addition, the findings will be promoted through papers in peer reviewed journals, conferences, and professional networks as well as through local Healthwatch.

**Study Title**	SHINE Study: Developing an intervention for **s**afe **h**ospital **in**sulin us**e** for older/frail adults with diabetes undergoing a surgical admission to hospital.
**Internal ref. no. (or short title)**	**SHINE Study**: Developing an intervention for **S**afe **H**ospital **IN**sulin us**E**
**Protocol Version number and Date**	v.1.0 - 12/12/2023
**IRAS Number**	334058
**REC Reference**	
**Chief Investigator**	Dr Kirsty Winkley
**Study Coordinator & Lead Researcher**	Mrs Christina Lange Ferreira
**Study Sponsor**	King's College London
**Funder**	Foundation of European Nurses in Diabetes<br>Herefordshire and Mid-Powys Trust Fund
**Medical Condition or Disease under investigation**	Diabetes
**Study Design**	Qualitative Study: Development phase of complex intervention using a Co-design approach using design thinking.
**Study Participants**	People with diabetes and their carers/family member<br>Healthcare professionals & non-clinical staff working in NHS
**Planned Size of Sample (if applicable)**	Up to 15 patients/their carers<br>Up to 25 HCP/non-clinical staff working in NHS
**Follow up duration (if applicable)**	N/A
**Planned Study Period**	12 months
**Research Question/Aim(s)**	The **overarching research question** is:<br>*What components of hospital insulin use can be identified to develop a conceptual model/toolkit to support more consistent system-based insulin safety/incident identification, classification, exploration and learning in older/frail adults with diabetes undergoing a surgical admission?*<br>The overall aim of this study is to contribute towards hospital insulin safety efforts by drawing together patients and staff in a co-design process to develop a conceptual model of a complex intervention to support the insulin safety review process, to improve the experiences and safety of patients with diabetes treated with insulin undergoing a surgical hospital admission and reduce insulin errors.<br>The collaborative process will incorporate the views and experiences of patients/their carers or family members, healthcare professionals (HCP), underpinning theory and relevant complex socio-technical components of the hospital environment.

	**Specific Aims/Objectives to be addressed at different phases of project in this application**:<br>• To explore and understand the experiences and perspectives of older/frail adults with diabetes undergoing a surgical admission regarding insulin safety in hospital & insulin safety incidents learning and response.<br>• Identify important factors in insulin safety & errors review process from the patient perspective.<br>• To explore and understand perspectives of HCP/staff engagement and ownership of diabetes care and insulin safety.<br>• To explore and understand the experiences and perspectives of NHS staff regarding insulin safety incidents/errors and learning and response.<br>• To identify important factors in safe insulin use in older/frail adults with diabetes undergoing surgery from the HCP perspective.<br>• To identify content, associated resources and implementation aspects for potential intervention and toolkit development to support more consistent system-based insulin safety exploration, learning and response.<br>• To identify how RESILIENT framework can be used in practice in the context of system-based insulin safety exploration, learning and response<br>• To identify through a co-design process, key components and attributes of future intervention/toolkit to support more consistent compassionate system-based insulin safety/incident identification, classification, exploration, learning and response and to support identification of vulnerabilities and opportunities to strengthen hospital system insulin use and prevent insulin errors in older/frail adults with diabetes undergoing a surgical admission.
**Main Inclusion criteria**	**Service user/Patient inclusion criteria**<br>• Are able to give informed consent<br>• Age ≥ 65 years OR frailty identified/documented on admission<br>• Patient with a diagnosis of diabetes before their hospital admission and treated with insulin therapy before and during hospital stay<br>• History of hospital admission for major surgery with minimum 1 night hospital stay post-surgery at study site selected surgical wards: within the last 9 months.<br>• Fluent English speaker. Portuguese speaking patients can be included at interview stage<br>**Family member/carer inclusion criteria**<br>• Family members such as older/frail patients' children, siblings, spouses, friends or carers who speak and understood English and are involved in the usual diabetes care/insulin management of eligible patients may be considered for inclusion if invited by the patient/service user.<br><br>**NHS Staff inclusion criteria**<br>• Clinical and non-clinical staff involved in the hospital care/transfer of care/insulin incident or safety review of older/frail adults with diabetes undergoing surgical admission at the study site designated wards<br>• Working full or part time at the study sites (for a minimum of 3 months)
**Study Site**	Surgical wards (including general surgery and frailty trauma & orthopaedic wards) in one NHS Hospital Trust in the West Midlands: Wye Valley NHS Trust (Hereford County Hospital).

FUNDING AND SUPPORT IN KIND

FUNDER(S)<br><br>(Names and contact details of ALL organisations providing funding and/or support in kind for this study)	FINANCIAL AND NON FINANCIALSUPPORT GIVEN
Foundation of European Nurses in Diabetes<br>Registered Charity no. 1134995<br>Company limited by guarantee<br>Registered in England no. 7114723<br>Registered Office: 37 Earls Drive, Newcastle on Tyne, NE157AL, UK<br>Tel: +44 (0)2088766122	**PhD Fees and salary**
Herefordshire and Mid-Powys Trust Fund<br>42 Rural Enterprise Centre<br>Vincent Carey Road<br>Rotherwas<br>Hereford<br>HR4 0LQ<br>Telephone: 01432345703	**Patient and staff research involvement costs**: up to 600 pounds for participation thank-you vouchers
**TBC**<br><br>**Grant award applications awaiting outcome**	**King's College London Centre for Doctoral Studies** small grant of up to £1,000 to deliver a public engagement activity as part of their research. Application for funding to cover patient travel costs, light refreshments at co-design events and Graphic designer input to develop an infographic of patient priorities in hospital insulin safety as told by patients.<br><br>**Novo Nordisk Clinical Project Fund Award** applied for to cover professional transcription costs. If unsuccessful, the lead researcher will carry out the transcriptions.

ROLE OF STUDY SPONSOR AND FUNDER

The sponsor is responsible for securing the arrangements to initiate, manage and finance a study.

In practice this means satisfying itself the research protocol, research team and the research environment have passed appropriate scientific quality, satisfying itself that the study has ethical approval before it begins; satisfying itself that arrangements are kept in place for good practice in conducting the study, and for monitoring and reporting, including prompt reporting of suspected unexpected serious adverse events or reactions and ensuring arrangements are in place for insurance and/or indemnity to meet the potential legal liability of the sponsor(s) for harm arising from the research.

The sponsor, King's College London (KCL), will take primary responsibility for ensuring that the design of the study meets appropriate standards and that arrangements are in place to ensure appropriate conduct and reporting. KCL takes responsibility for arranging the initiation and management of this research, and for ensuring that appropriate standards, conduct and reporting are adhered to regarding its facilities and staff involved with the project.

Decisions relating to data analysis and interpretation, manuscript writing, and dissemination of results will be made by the research team at King's College London University.

The funder will be kept informed by the team and made aware of results prior to publication but published outputs will contain a disclaimer reflecting the fact that results and related interpretation are those of the researchers and not the funder.

ROLES AND RESPONSIBILITIES OF STUDY MANAGEMENT COMMITTEES/GROUPS & INDIVIDUALS

- **Supervision arrangements as part of Doctoral Programme**

- **Patient & Public Involvement Group**

Healthwatch Herefordshire will support with PPI involvement, including:

Review and comment of study service user facing paperwork
Dissemination of call to recruitment
Offer pathways into particular user groups/stakeholders for consultation and dissemination purposes
Dissemination of findings through their networks
Highlight possible areas of collaboration with other projects

PROTOCOL CONTRIBUTORS

This protocol was developed by the research team with support from King's College London University and Wye Valley NHS Trust. This protocol is for Stage 2 and 3 of a three stage project forming a doctoral research project. Dr Sara Donetto from Brighton and Sussex Medical School kindly reviewed the protocol and offered comments and feedback from a methodological perspective.

**KEY WORDS:**	Insulin safety
	Diabetes
	Co-Design
	Inpatient Safety
	Older adult
	Frailty

STUDY FLOW CHART

Doctoral Research Study Diagram

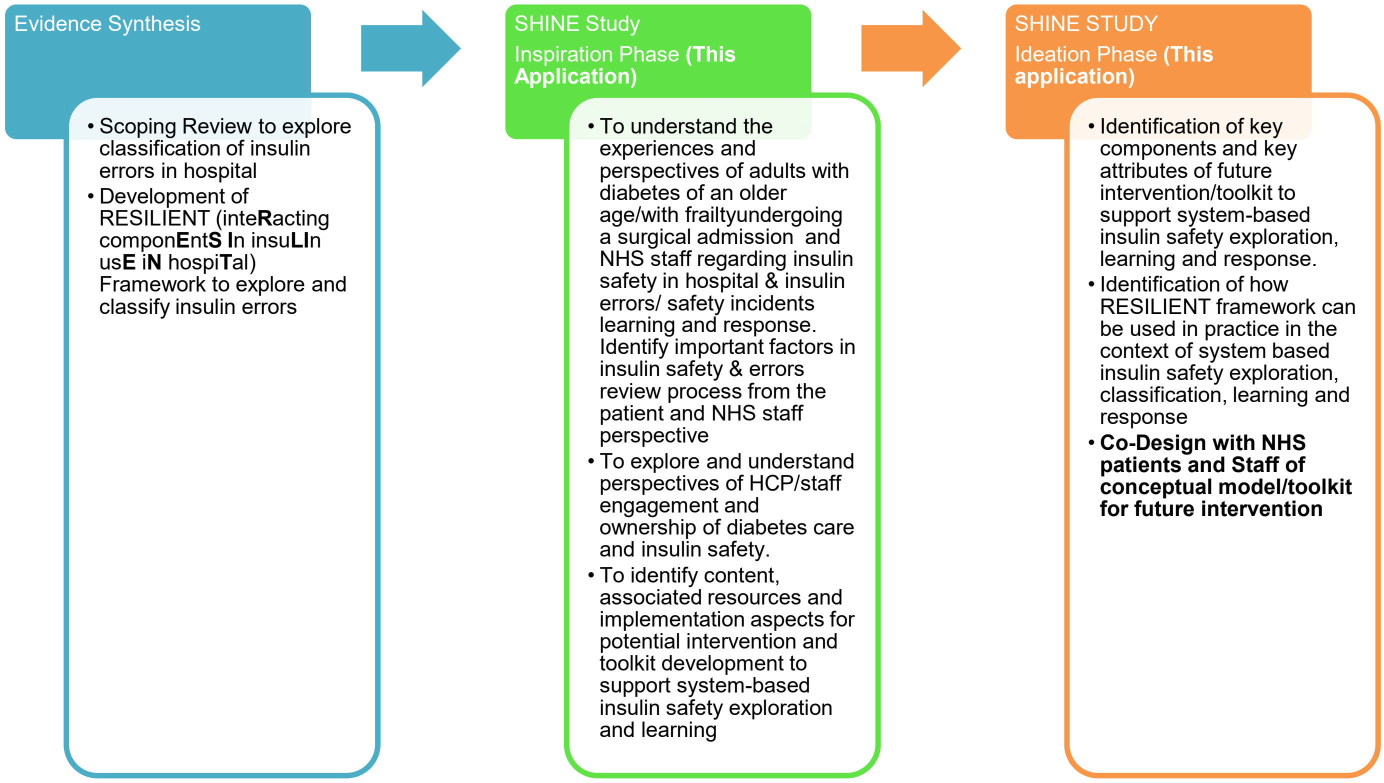

SHINE Study Flowchart

Setting up → Local Engagement, relationship building, timelines, Recruitment &informed consent process for observations, interviews, workshops → Approach up to 15 patients with experience of hospital admission in last 2-24 weeks and relevant staff participants → Informed consent

Phase	Data collection point	Participants	Activities	Recruitment and informed consent process
**Inspiration Phase**	**Observation**	HCP/Staff	Non-participatory observation of insulin incident/safety review meeting(s)	Informed by ethical and consent process<br>Staff can exit study at this point if desired
	**Interviews**	Older/Frail adults with experiences of surgical admission to hospital	Audio recorded Semi-structured interviews	Informed by ethical and consent process<br>Patients can exit study at this point if desired.
	**Interviews**	HCP/staff at NHS hospital	Audio recorded Semi-structured interviews	Informed by ethical and consent process<br>Staff can exit study at this point if desired.

Initial analysis of *Inspiration Phase* data in preparation for *Ideation Phase &*

Engagement activities between inspiration and ideation phases

Further patient & staff recruitment if required

Phase	Data collection point	Participants	Activities	Recruitment and informed consent process
**Ideation Phase: Substage A:** Parallel workshop with each participants group	**Workshop with patients** N=1	Older/Frail adults with experiences of surgical admission to hospital	Audio recorded workshop	Informed by ethical and consent process<br>Patients can exit study at this point if desired.
	**Workshop with staff N=1**	HCP/staff at NHS hospital	Audio recorded workshop	Informed by ethical and consent process<br>Patients can exit study at this point if desired.
**Ideation Phase: Substage B:** Integrated co-design workshops	**Integrated co-design workshops (n= minimum 2)**	Older//Frail adults with experiences of surgical admission to hospital & HCP/staff at NHS hospital	Audio recorded workshop	Informed by ethical and consent process<br>Patients & Staff can exit study at this point if desired.

NHS Wye Valley NHS Trust NHS Health Research Authority King's College London

Study GANTT Chart

		Jan 24	Feb 24	Mar 24	April 24	May 24	June 24	July 24	Aug 24	Sept 24	Oct 24	Nov 24	Dec 24
		REC, R&D AND HRA Approvals approved before study commences											
Setting up		●											
Inspiration Phase													
Non-participant observations		●	●	●									
Interviews with patients		●	●	●	●	●							
Interviews with staff			●	●	●	●							
Data analysis			●	●	●	●							
Initial analysis of Inspiration Phase to prepare for Ideation Phase							●	●					
Ideation Phase													
Ideation Phase: Part A	Workshop with patients									○			
	Workshop with Staff									○			
Ideation Phase: Part B	Integrated co-design workshops											○	○
Data analysis										○	○	○	○

STUDY PROTOCOL
SHINE Study: Using a co-design approach to develop a conceptual model/tool-kit of an intervention to support hospital system-based insulin safety and learning review for **S**afe **H**ospital **IN**sulin us**E** (SHINE) in older/frail adults with diabetes undergoing surgical admission in hospital

1. BACKGROUND

Insulin and its importance in diabetes management

Insulin, a hormone essential to life, plays a key role in regulating glucose metabolism (1). Diabetes, estimated to affect 4.9 million people in the UK (2), is a serious chronic metabolic disease characterised by hyperglycaemia or high glucose levels. It is an umbrella term for different types of diabetes but in all of them there are defects in the secretion and/or action of insulin in the target tissues (3,4). Insulin therapy is a crucial part of treatment for many people with diabetes (5,6).

Insulin is an injectable therapy which requires refrigeration; there are several different types of insulin and insulin regimens with different onset, peak, and duration of action. Some people take once daily injections, others multiple injections (7,8). It is very challenging to attempt to reproduce the physiological insulin profile via administration of insulin injections; insulin treatment requires high levels of treatment individualisation regarding regimen choice, dosing, dose adjustments. Most of the time this therapy is self-managed by the patient (8).

Insulin: a high-risk and time critical medication

Insulin is considered a high-risk and time critical medication, due to its narrow therapeutic index and level of harm that can happen in the event of an error: life threatening seizures and coma due to hypoglycaemia (low glucose level) which can result in long-lasting neurological damage and even death; life threatening ketoacidosis or hyperosmolality resulting from hyperglycaemia (high glucose level) (9). Insulin errors are common and seen as a complex problem. They can occur at any stage of the complex and multi-step medication use process which includes appropriate prescribing and risk assessment, medication review, supply, dispensing, storage, preparation, administration, disposal, communication and patient engagement, medication reconciliation at care transitions; all these underpinned by person centred care and shared decision making (10–12).

Diabetes and insulin care in hospital

In the UK, one in six people occupying a hospital bed has diabetes, and in some hospitals the prevalence is higher (13).

Hospitals are recognised as complex adaptive systems (14). The hospital environment adds to the complexity around insulin use with its multiple interacting components: patient, healthcare professional (HCP) and contextual factors to consider. For example, at a patient level, an individual's physiological response to stress and illness can impact on glucose control, changes in appetite or ability to eat, insulin requirement and ability to self-manage. At a contextual level there are factors such as changes in usual meal times and type of food available, new or changed treatments (15). HCP factors, such as knowledge gaps, poor communication, errors in documentation, busyness and tiredness use of discouraged practices amongst others have been identified as impacting on insulin use (11,16–18).

Several national and local initiatives have been designed to improve insulin safety in UK hospitals, amongst them: Think Glucose, a national programme to improve diabetes care, national audits (13) and

local audits (17,19) to support identify areas for enhanced insulin safety and guide service improvement; insulin specific drug charts (20), electronic prescribing (13), increased training for staff (18,21,22), involvement of pharmacists (23,24), encouragement of self-management of insulin (25), e-learning modules for staff (26), amongst others, with varying degrees of success (27). Many of these interventions were not theory-driven or designed in collaboration with service users.

Data from the latest National Diabetes Inpatient Audit (NADIA), an annual snapshot audit of diabetes care in inpatient areas in England and Wales which ran from 2010-2019, shows a persistent problem despite decades of insulin safety interventions: 40% of insulin-treated inpatients had one or more insulin error (NHS Digital 2020). The CQC report on medicines in health and social care identifies insulin as a high-risk medication in the hospital environment; frequent notifications received about insulin related errors with adverse consequences reported (11). Insulin use in hospital is a source of anxiety and distress for people with diabetes in hospital, who many times are reported to not feel safe (28,29).

Lack of clarity and consistency in identifying, reporting, exploring and classifying insulin errors

A scoping review was undertaken to classify the types of insulin errors occurring in hospital and identify potential interacting components in insulin use/insulin error. A reproduceable search of 3 online databases (Medline, CINAHL and Embase) as well as focused search of grey literature was undertaken.

Records which identified, explored or examined insulin errors examining insulin errors in adult inpatients with diabetes, published from year 2000-May 2023 in English language were considered for inclusion. Studies were not excluded on the basis of study design. From 3391 records identified, 117 records were included in the final synthesis. The review identified a range of insulin use errors at all stages of the medication use process and significant variability in terms of exploring, defining, classifying and reporting insulin errors. Given the lack of consistency in exploration of insulin errors, through an iterative process a RESILIENT (inte**R**acting compon**E**nt**S** in **I**nsu**LI**n us**E** i**N** hospi**T**al) framework (Figure 1) was developed and applied to the analysis and mapping of insulin errors and potential interacting components in insulin use. Further work is needed to understand how RESILIENT Framework can be used in practice.

The review identified a lack of system-based exploration of insulin use/insulin errors in hospital as well as lack of patient voice/participation in the design of insulin safety interventions. Further work on developing system-based learning and response regarding insulin safety incidents could support prospective system-based resilience strengthening to improve insulin safety.

Study Population: focusing on the surgical admission

The patient population of interest to the study are older/frail adults with diabetes, who have undergone major surgery with a minimum of 1 overnight stay in hospital post-operatively.

It has been well established that good glycaemic control can improve outcomes for patients with diabetes undergoing surgical procedures, and insulin use often plays a key role in this (30–32). Patients undergoing surgery often develop stress hyperglycaemia. Post-operative hyperglycaemia is associated with poorer outcomes such as risk of infection, re-intervention and death (30,33).

The choice to focus on the surgical admission is because several ‘high risk factors’ for insulin errors along the peri-operative journey have been identified and described in national reports such as NADIA, National Confidential Enquiry into Patient Outcome and Death (NCEPOD) and the Getting it right first time (GIRFT) (13,34,35). On reviewing the care of patients with diabetes undergoing peri-operative care, NCEPOD report found insulin reported errors to be the most common in regards to diabetes medication

errors (34). From a contextual perspective, patients undergoing a surgical admission will experience transitions of care within the hospital (eg. admission, ward, theatre, recovery, ward) and on discharge, input from multiple healthcare professional teams and potential high-risk situations such, changes to insulin doses pre surgery, starvation periods for procedures, potential IV insulin use; post-operative pain and physical restrictions amongst others. These are identified as key action areas in RESILENT framework, recognising the higher risk for insulin error.

The National Diabetes Inpatient Audit undertaken in 2019 highlighted that only 2 out of 5 surgical inpatients with diabetes had their glucose appropriately monitored at all recommended stages of the peri-operative pathway (13). Glucose monitoring and management is essential for safe insulin management. Furthermore, patients admitted under surgical specialties have been identified as having a higher risk of developing hospital acquired Diabetic Ketoacidosis (DKA) (36) and this is associated with mis-management of insulin during admission. Recognising the implementation gap in recommendations and care a multidisciplinary guideline for perioperative care for people with diabetes mellitus undergoing elective and emergency surgery has been developed (31), and nationally there is a focus on improving the care of people with diabetes undergoing surgery.

Study Population: focusing on older/frail adult with diabetes undergoing a surgical admission

The choice of older adults or frail adults with diabetes as the population in this study is because they are at greater risk and are more vulnerable in the context of insulin errors. Diabetes in older age is frequently associated with other features such as co-morbidities, frailty, polypharmacy, and a number of potential deficits: cognitive, physical, nutritional, communication (37). Frailty has been associated with greater risk of post-surgical complications and increased length of stay (38).

The impact of frailty and the ageing process has implications in diabetes management and risks, for instance hyper or hypoglycaemia. Osmotic symptoms may be less apparent in older frail people, putting them more at risk of hyperglycaemic emergencies such as DKA or Hyperosmolar Hyperglycaemic State (HHS). Symptoms of hypoglycaemia may also be impaired with loss of autonomic symptoms of hypo and delayed recovery post hypoglycaemia, making them more vulnerable to experiencing severe hypoglycaemia (38). In the NCEPOD report into the care of people with diabetes who underwent a surgical procedure, the median age was 69 years, and 38.6% of patients were identified as vulnerable or frail.

As diabetes is a disease which requires such high levels of self-management, older age and frailty can impact on this significantly. Physical or cognitive dysfunction may impact on ability to self-manage diabetes treatments and lead to the need for third party administration of insulin therapy in hospital. Changes in appetite and mobility can affect treatment requirements and glucose control leaving them more vulnerable to hypoglycaemia or hyperglycaemia, which they may be less symptomatic of anyway, so these can be harder to detect. Deficits in cognition and communication can add further challenges. Individualized care planning are very important in this vulnerable group of patients (37) as many of these characteristics increase the risks for insulin error in hospital.

Evidence has shown errors occur at multiple stages of the peri-operative journey, and recurrent types of errors persist. Having a deeper understanding of how HCPs engage with and view the insulin use process and the insulin review/learning from incidents through the patient peri-operative journey is an important aspect of the development of a tool to target these aspects and contribute towards the efforts for improvement in the care and hospital experience of people with diabetes undergoing surgery.

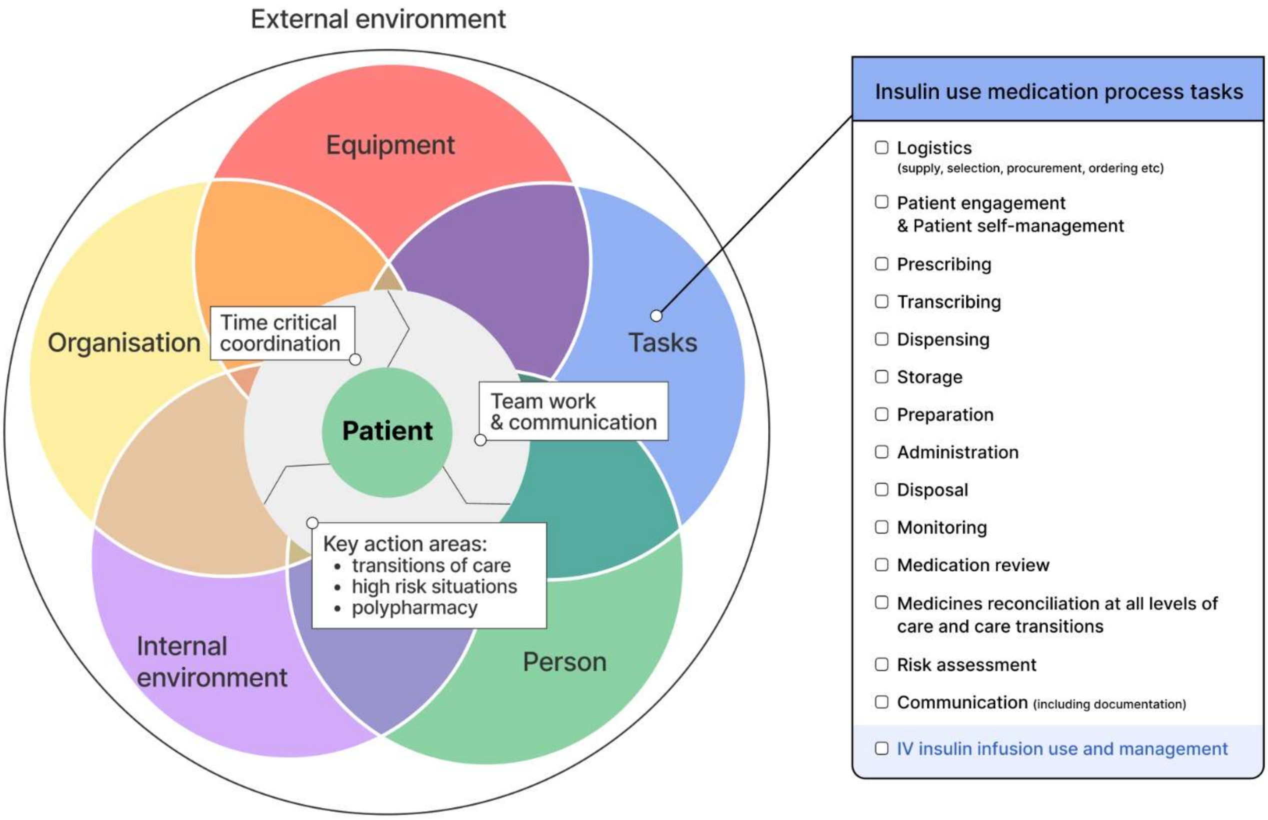

Figure 1 RESILIENT (inteRacting componEntS in InsuLIn usE iN hospiTal) framework infographic

2. RATIONALE

The evidence presented above in the background section demonstrates that insulin errors in hospital still occur relatively frequently in inpatients with diabetes and have a potential risk for severe patient harm. Despite decades of interventions, and some improvements, patient safety issues around insulin use still persist (11,13). The complexity around hospital insulin use has been presented highlighting particular risks with older/frail adults undergoing a surgical admission. In this context insulin errors are a complex problem to address; continual concerted multi-pronged efforts to improve patient safety are required.

The scoping review undertaken as part of this project and discussed in the previous section identified a lack of system-based exploration of insulin use/insulin errors in hospital as well as lack of patient voice/participation in the design of insulin safety interventions. Further work on developing system-based learning and response regarding insulin safety incidents could support prospective system-based resilience strengthening to improve insulin safety.

Traditional approaches to incident review tended to be based around linear explanation of events. However, in complex problems and within complex adaptive systems with multiple interacting components this approach will only get you so far; unless a system approach is taken which considers multiple interacting components involved, opportunities for enhancing the system resilience will be, arguably, missed (14,39).

The NHS is changing its approach to patient safety and incident review with its new Patient Safety Incident Response Framework (PSIRF) and toolkit (40) which is launching over the course of 2023.

Compassionate engagement and involvement of those affected by incidents is central to PSIRF, as is a systems-based exploration of events aimed at learning and strengthening of systems. This will require a significant cultural shift, as well as development of new processes and tools.

Within this backdrop and recognising the complexity around hospital insulin use, developing a tool/toolkit that supports more consistent system-based approach to exploring hospital insulin use and errors, arguably enables a better understanding of system use of insulin and identifying touchpoints and patterns of interactions where system resilience could be enhanced, thereby improving insulin safety and inpatient experience for patients. Ensuring the patient and end users are involved in the process is considered to be essential.

Consultation with people with diabetes and their carers through one-to-one conversations, discussion in local diabetes UK group meeting, diabetes patient group and engagement with Healthwatch have confirmed that people with diabetes suffer anxiety and have experienced insulin use related errors during their hospital stay. There is disbelief at the type of errors that happen and more research in this area is considered very important. People with diabetes want to feel increased confidence and safety in the care professionals and environments looking after them, at a time when they feel particularly vulnerable.

This study would enable a valid contribution towards insulin safety for older/frail adults with diabetes undergoing surgery by supporting decision-making to avoid insulin errors in hospital for this population.

3. THEORETICAL FRAMEWORK

Safe insulin use in hospital has already been presented as a complex problem with multiple interacting components. Complex interventions are characterised by having multiple interacting components, not only between the components of the intervention itself but also of the intervention components with and within the context in which it is delivered (41). Therefore, careful development of complex interventions is required, to increase the chances of the intervention being adopted and having desired effects when implemented (42). The joint National Institute for Health Research (NIHR) and the UK Medical Research Council (MRC) new framework for developing and evaluating complex interventions identifies four phases of complex intervention research: development or identification of an intervention, feasibility, evaluation and implementation (41). This study would form part of the development phase of a complex intervention.

As insulin is such a highly individualised treatment where self-management/carer support is so key and with insulin errors in hospital affecting their experience of hospital stay it becomes crucial to heavily involve the patient and the staff in the decision making in the development of this intervention, to listen to their narratives, experiences and insights and co-design the intervention together, aspiring to produce better experiences of care. This being the case a 'partnership' approach to intervention development using Co-Design appears adequate to this study (42). From a methodological perspective this study draws from Participatory Research and will use a Co-Design approach using design thinking.

Design thinking draws from human centred design and a creative mindset and generates an approach to developing creative ideas and solutions involving key stakeholders and end users in the process. Design Thinking has successfully been applied to medicine and health care sector to support the development of products and practices that address everyday challenges (43). Design Thinking does not follow a rigid methodological process and acknowledges that design does not always follow a linear process but identifies 3 phases which the design process involves: Inspiration, Ideation and Implementation; characteristics of these 3 phases are briefly described in Table 1. A range of data

collection methods can be used depending on study priorities and can include observations, interviews, focus groups, workshops (44).

This proposed study would focus on the Inspiration and Ideation phases. Implementation phase would be a future study.

**Phases of Design Thinking**	**Characteristics**	**Core Principles**
**Inspiration**<br><br>*(This study)*	Engagement with key stakeholders<br>Empathise: fostering a culture of care and understanding/sharing in the experiences of patients, carers and staff<br>Framing design challenge<br>Learning and exploration<br>Relationship building	Empathy, Optimism, Iteration, Creative Confidence, Making, Embracing Ambiguity, and Learning from Failure
**Ideation**<br><br>*(This study)*	Share learning from *Inspiration* phase<br>Sense making<br>Identifying opportunities for design<br>Generation of ideas; some to keep some to discard<br>Prototype development<br>Multiple iteration and refinement cycles	
**Implementation**<br><br>*(Future study)*	Testing prototype developed in *Ideation* phase<br>Partnerships<br>Monitoring, feedback and evaluation	

Table 1 Phases of Design Thinking and characteristics (44)

This study will draw from Complex Systems thinking, Safety 2 and Resilient Healthcare principles in the development of the intervention (14,39). Safety 2 and Resilient Healthcare principles will be used in the theory-based analysis of this study. Theory based analysis using Safety 2 approach has previously been incorporated into Experience Based Co-Design (EBCD) process (45). Using safety 2 and resilient healthcare principles to learn from incidents has been found to focus attention on ways of strengthening systems prospectively (46). Further theoretical lenses may be used in this study, guided by the data and priorities emerging from the research process. **Erro! A origem da referência não foi encontrada.** outlines in a visual way the key theoretical concepts that frame this study.

VISUAL REPRESENTATION OF THE THEORETICAL FRAMEWORK INFORMING THE SHINE STUDY

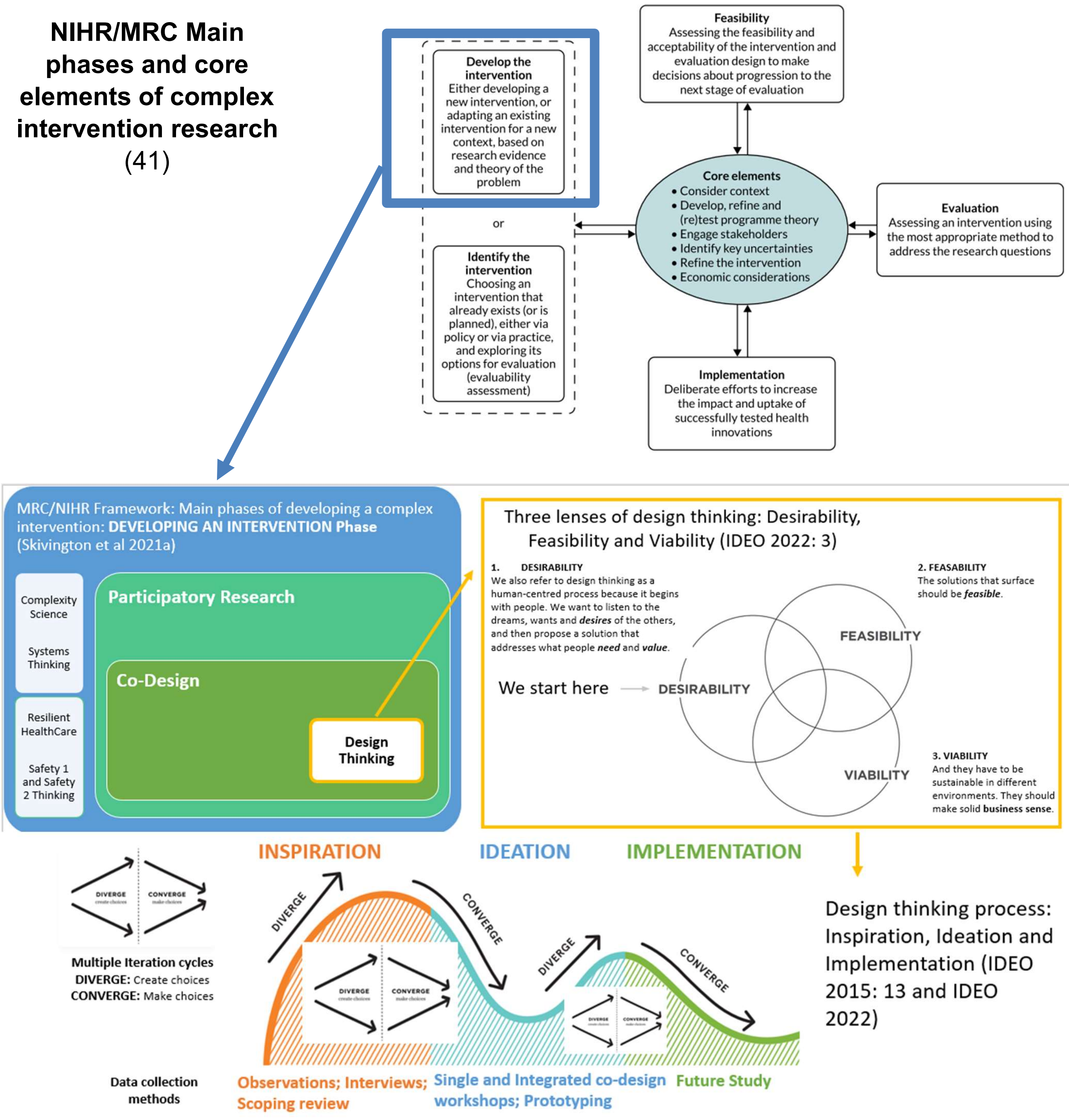

Figure 2 Visual representation of theoretical framework informing this study

4. RESEARCH QUESTION/AIM(S)

The **overarching research question is:**

What components of hospital insulin use can be identified to develop a conceptual model/toolkit to support more consistent system-based insulin safety/incident identification, classification, exploration and learning in older/frail adults with diabetes undergoing a surgical admission?

The overall aim of this study is to contribute towards hospital insulin safety efforts by drawing together patients and staff in a co-design process to develop a conceptual model of a complex intervention to support the insulin safety review process, to improve the experiences and safety of patients with diabetes treated with insulin undergoing a surgical hospital admission and reduce insulin errors.

The collaborative process will incorporate the views and experiences of patients/their carers or family members, healthcare professionals (HCP), underpinning theory and relevant complex socio-technical components of the hospital environment.

4.1 Objectives

Specific Aims/Objectives to be addressed at different phases of project in this application:

- To explore and understand the experiences and perspectives of older/frail adults with diabetes undergoing a surgical admission regarding insulin safety in hospital & insulin safety incidents learning and response.
- Identify important factors in insulin safety & errors review process from the patient perspective.
- To explore and understand perspectives of HCP/staff engagement and ownership of diabetes care and insulin safety.
- To explore and understand the experiences and perspectives of NHS staff regarding insulin safety incidents/errors and learning and response.
- To identify important factors in safe insulin use in the older/frail adult with diabetes undergoing surgery from the HCP perspective.
- To identify content, associated resources and implementation aspects for potential intervention and toolkit development to support more consistent system-based insulin safety exploration, learning and response.
- To identify how RESILIENT framework can be used in practice in the context of system-based insulin safety exploration, classification, learning and response.
- To identify through a co-design process, key components and attributes of future intervention/toolkit to support more consistent compassionate system-based insulin safety/incident identification, classification, exploration, learning and response and to support identification of vulnerabilities and opportunities to strengthen hospital system insulin use and prevent insulin errors in older/frail adults with diabetes undergoing a surgical admission.

4.2 Outcome

To develop, through a co-design process, a conceptual model/toolkit of a future intervention to support more consistent compassionate system-based insulin exploration, learning and response and to support identification of vulnerabilities and opportunities to strengthen hospital system insulin use and prevent insulin errors in older/frail adults with diabetes undergoing a surgical admission.

To achieve part of the development phase of developing an intervention in partnership with patients with diabetes/their carers and healthcare professionals, to enhance insulin safety for older/frail adults with diabetes undergoing surgery.

5. STUDY DESIGN and METHODS of DATA COLLECTION AND DATA ANALYIS

5.1. Study design

As previously outlined in section 3, this study draws from the MRC & NIHR Framework for the development and evaluation of complex interventions(41,42).

From a methodological perspective this study draws from Participatory Research and will use a Co-Design using Design Thinking. A variety of data collection methods will be used and further described below.

5.2. Methods of data collection

Data will be collected employing various methods. Observation, interview and co-design data will be collected and analysed by the lead researcher.

To describe the study sample, patient participant demographic profile will be collected such as age, gender, ethnicity, marital status, highest level of education, occupation, usual place of residence, type of diabetes diagnosis, duration of diabetes, diabetes treatment, type of insulin treatment, device used and doses, method of glucose monitoring, level of independence regarding insulin self-management, surgical specialty admitted under, duration of hospital admission, number of hospital admissions in last 12 months, type of surgical ward during hospital admission, discharge destination.
Staff demographic profile such as age, gender, ethnicity, job role, length of time in current role, length of time working in NHS will be collected.
Participants' identity will be anonymised by allocating study identifier codes.
Each data collection method is further described below.

5.2.1 Observation of incident review meeting (Inspiration Phase)

Observations will allow the lead researcher to explore features of day-to-day activity and ways that insulin incident/safety & learning review is manifest in the hospital site which the participants may view as ordinary or unremarkable. It allows the researcher to see naturally occurring situations of interactions, communication, behaviours and relationships within a context.
The aim of these observations is to have a sense of what things look like in practice so that the context the staff and patients are in is understood ahead of the interviews and co-design interactions with patients and staff. The aim of the observations is not to generate data for in depth analysis of the observation notes as there would not be time for this within the study.
The observations would take place at the beginning of the Inspiration phase as part of local exploration and engagement and identifying evidence and developing theory. Attempts will be made to observe a minimum of 2 meetings, however this will be influenced by organizational and operational capacity to accommodate this.
Contemporaneous or post observation field notes will be collected by the lead researcher guided by an observation tool. The lead researcher would be a non-participant observer to minimise interference of clinical care discussions/interactions and to facilitate ease in recording field notes. There is an acknowledgement that as participants are aware they are being observed, behaviours may change. The purpose of the observations will be framed as an opportunity to learn more about the insulin incident and safety review process as part of a wider study seeking to better understand the experiences of patients and staff and identifying how to support insulin safety in hospital.
During the course of observations, the lead researcher may ask questions to staff, prompted by an activity, behaviour or personal reaction. These informal conversations would not have an interview

schedule and would be undertaken to gather greater insight into why people behave the way they do or perform tasks in the order that they do. They allow the lead researcher to gather opinions about what participants thought or think about events that have just been experienced. Either contemporaneous or as soon as possible post event field notes of these conversations will be collected by the lead researcher.

5.2.2 Semi-structured interviews (Inspiration Phase)

Semi-structured, in-depth interviews will allow the researcher to set the topics/themes that will be explored in the interaction but the participant's responses will determine the forms of information that are generated. The observations and relevant literature review will help inform development of a topic guide which the lead researcher will use and ask probing questions to further explore responses. A topic guide will be used in these semi-structured interviews and the use of probing questions will be employed to explore topics in depth. These interviews will aid the researcher to gain greater insight into participants subjective experiences, perspectives and support greater understanding and interpretation of events.

Audio-recorded interviews with patients post hospital discharge and with staff will be conducted in a private single hospital room or virtually depending on participant preference. Interviews can last up to an hour.

5.2.3 Engagement activities between inspiration and ideation phases

Between inspiration and ideation phases some engagement activities will take place to continue engaging with participants, keeping momentum and to potentially consult/discuss early thoughts from interviews/workshops in preparation for workshop events. Amongst these telephone calls, email, online whiteboard or other means that are appropriate and accessible to the participants may be used.

5.2.4 Co-design workshops (Ideation Phase substage part A & B)

These workshops sit within the *Ideation phase* which uses the findings from the *Inspiration phase* as a springboard for the co-design groups to generate ideas, possible solutions and prototypes. The prototype or conceptual model/toolkit of an intervention to support hospital system-based insulin safety/incident exploration, learning and response will be produced at the end of this phase.

There will be a commitment to giving everyone a voice and to take all voices seriously.

The workshops will be informed by published tool-kits to support co-design process (44,47). Activities such as empathy maps, brainstorming, journey map, storyboard etc are likely to employed.

The lead researcher will be present in all events, carry out the facilitation and moderation of the workshops, supported by a member/s of the diabetes team who will provide general support with note taking, planning and management of the workshop events. Events will be audio-recorded. Materials and 'presentations' created through activities used in the workshops will also be included as data to be analysed. If participants are unable to attend the co-design workshops but would still like to be involved in the co-design process this will be facilitated through telephone calls, email, online whiteboard or other means that are appropriate and accessible to the participants may be used.

The lead researcher may also collect field notes to record interactions, comments, dynamics, feelings and relationships as the participants take part in the workshop & co-design events.

There will be 3 different types of workshops: single workshop with patients (ideation substage A); a single workshop with staff (ideation substage A) and then a minimum of 2 integrated workshops with

patients and NHS staff (ideation substage B). Table 2 describes the workshops, participants and aims of the different workshops.

Phase	Type of workshop	Who will attend	Aim of workshop
**Ideation Phase**: Substage A: **Parallel workshop with each participants group**	Single workshop with patients/their carers	Patients who were interviewed will be invited to attend this workshop.	○ Presenting the findings from the Inspiration *Phase amongst patients*<br>○ Validation of findings<br>○ Prioritisation of identified challenges or areas amenable to intervention development<br>○ Establishment of relationships and connections for future co-design workshops
	Single workshop with NHS staff	NHS staff who were interviewed will be invited to attend this workshop.	○ Presenting the findings from the *Inspiration Phase amongst NHS staff*<br>○ Validation of findings<br>○ Prioritisation of identified challenges or areas amenable to intervention development<br>○ Establishment of relationships and connections for future co-design workshops
**Ideation Phase**: Substage B: **Integrated co-design workshops**	Between 2 and 4 Integrated patient-staff co-design workshops	Patients who were interviewed will be invited to attend. NHS staff who were interviewed will be asked for expression of interest to attend these workshops<br><br>Patients and NHS staff will be active participants in these workshops and may also be working alongside each other in smaller groups within the workshop.	○ Present the findings from Inspiration phase<br>○ Present the priorities from the parallel workshops with each participant group *(ideation phase substage A)*<br>○ Prioritise areas for development<br>○ Generation of ideas and preliminary solutions and through an iterative process build prototypes of conceptual model/toolkit for intervention<br>○ The final workshop will be to present the final conceptual model/toolkit of intervention prototype and elicit feedback/final refinement prior to being ready to proceed to testing phase in a future study

Table 2 Types of workshops in SHINE study, participants and aims of workshops

5.2.5 Summary of service user/patient study activities

The summary of patient study activities, average time for visit and who will be conducting the activities can be seen in Table 3.

Activity	Average Time for contact/visit	Who will conduct and description of activity
First contact with potential participant	10 minutes	Clinical teams identify and make initial approach to ask patient if they are interested.<br>Patients may also self-refer following call to recruitment.
Patient information & screening	Up to 30 minutes	If the patient agrees to be contacted, the lead researcher will:<br>1. Introduce/brief explanation of study<br>2. Provide a participant information sheet, study contact details and consent forms<br>**3.** Obtain permission to contact patient by phone and agree a date for the next meeting with the patient.
Reminder text or phone call	5 minutes	Lead researcher will text if possible or telephone the participant a day before the interview to remind of interview and to reduce incidents of no-show.
Informed consent & screening	Up to 15 minutes	Informed consent prior to research activities, supported by lead researcher. Participants will be encouraged to ask questions prior to providing consent.
Audio recorded interviews	Up to 60 minutes	Lead researcher will conduct audio-recorded interviews with the participant in a single room in the hospital or virtually depending on patient preference.
Reminder text or phone call	5 minutes	Lead researcher will text if possible or telephone the participant a day before the workshop to remind of workshop and to reduce incidents of no-show.
Patient workshop	120 minutes	This will take place in a meeting room in the hospital/designated site. The lead researcher will facilitate and moderate the event supported by an expert facilitator/s.
Reminder text or phone call	5 minutes	Lead researcher will text if possible or telephone the participant a day before each workshop to remind of workshop and to reduce incidents of no-show.
Integrated workshops	120 minutes	This will take place in a meeting room in the hospital/designated site. The lead researcher will facilitate and moderate the event supported by an expert facilitator/s. The number of meetings will be a minimum of 2 but may be more occurrences depending on the intervention refinement process.<br>The final workshop will be to present the final conceptual model/toolkit of intervention and elicit feedback/final refinement prior to being ready to proceed to testing phase in a future study.
Engagement activities between inspiration and ideation phases	Up to 30 minutes	Online or telephone communication facilitated by the lead researcher between inspiration and ideation phases some engagement activities will take place to continue engaging with participants keeping momentum and to potentially consult/discuss early thoughts from interviews/workshops in preparation for workshop events. Amongst these telephone calls, email, online whiteboard or other means that are appropriate and accessible to the participants may be used.

Table 3 Summary of patient study activities

5.3 Data analysis

Qualitative data will be analysed using thematic Framework Analysis (48). Framework analysis is a widely used analytical approach involving a systematic approach to organizing and analysing qualitative data within a framework of themes. Framework Analysis has been used in other co-design research (49).

Thematic framework analysis will use the following process (48) :

Step 1: Familiarization

Full immersion in the data. Active reading/listening and re-reading/ /listening of the data (transcriptions, notes) to become familiar with depth and breadth of the data, taking initial notes, ideas on the data. Key ideas and recurrent themes are listed.

Step 2: Identifying a thematic framework

Devising and development of the thematic framework (also referred to as index) is an iterative process. Drawing from apriori theoretical constructs; themes which have emerged from the literature review and the familiarization process: key issues, concepts and themes will be identified to which the data can be examined/referenced. This will help sift and sort the data within a thematic framework.

Step 3: Indexing/Coding

This phase relates to systematic application of the thematic framework to the data in textual form/transcripts. Data is read and indexed according to the thematic framework.

The framework will be systematically and continually revised until the data from all the transcripts is captured. A codebook of themes will be developed and modified by the researcher following an iterative process.

Step 4: Charting

Once the transcripts have been indexed a picture of the data as a whole is constructed. Charts with headings and subheadings are constructed. In the thematic approach charts are constructed for each key subject/theme area. The data will be entered into framework matrices for each theme in NVivo.

Step 5: Mapping and interpretation

During this phase, the key dimensions within the data will be identified, pulling together key characteristics and mapping, analysing and interpreting the data as a whole.

NVIVO 12 software will be used to aid the management of the data during analysis.

The collection, storage and disposal of the data during this study will strictly follow guidelines outlined in Data Protection Act (2018), General Data Protection Regulation (GDPR) (2018) and KCL Data Protection Policy which is found on: https://www.kcl.ac.uk/policyhub/data-protection-policy-2
Data will be anonymised using unique identifier codes. Encrypted devices will be used to record, interviews and workshop events. Recording files will be labelled with specific participant study number/code. If using external transcription services the recordings will be transferred via a secure website to an external agency within UK for transcription. Anonymised scripts will be downloaded from the secure agency website via the King's College London secure server. Audio recordings will be deleted as soon as analysis has been completed.

Regarding specific elements of each set of data:

5.3.1 Observations

Field notes will be digitally transcribed into ethnographic transcripts as soon as possible by the lead researcher.

5.3.2 Semi-structured interviews

Staff and patient interviews will be digitally recorded using 2 password protected audio recorders. Two devices will be used to mitigate for inadvertent failure of one of the devices. The data will be transcribed professionally using a King's College University approved transcriber or transcription will be undertaken by the lead researcher.

5.3.3 Co-design workshops

These events will be audio recorded using 2 password protected audio recorders. The aim is to record the main presentation and the whole-group discussions. The data will be transcribed professionally using a King's College University approved transcriber or transcription will be undertaken by the lead researcher.

Field notes will be taken by the lead researcher to capture comments, interactions, relationships, expressed feelings as the events occur. These will then be will be digitally transcribed into ethnographic transcripts as soon as possible by the lead researcher.

Pictures of the outputs created at the co-design events (post-it notes, mind-mapping etc) will used in the data analysis.

6 STUDY SETTING

The study will be conducted at one NHS Hospital Trust in the West Midlands: Wye Valley NHS Trust (Hereford County Hospital), focusing on surgical wards (including general surgery and frailty trauma and orthopaedic wards).

Patients admitted to designated surgical wards will be considered for inclusion. Clinical staff working along the perioperative journey of patients on those wards will be considered for inclusion, as well as staff involved in incident/safety/learning review concerning those wards.

Excluded settings:

The following ward settings will be excluded: Paediatric; Obstetric; A&E; Day case ward; Observation ward (if patients have been admitted for less than 24 hours); Day surgery unit patients (if patients have been admitted for less than 24 hours); Surgical short stay unit (if patients have been admitted for less than 24 hours); Palliative care centres; Community Hospitals; Mental Health; Intensive Therapy Unit; Medical wards

The decision to exclude the above wards aligns with the excluded areas from the National Diabetes Inpatient Audit (13) and ITU is excluded as patients would be unlikely to have recollection from their stay there, as well as it being a very specific clinical environment context. Medical wards are excluded as this study focuses on patient undergoing elective surgery. The surgical wards included in the study were suggested by the diabetes clinical team as they usually support the care of the majority of older/frail adults with diabetes undergoing surgical admission.

Serving a population of 186, 000 Hereford County Hospital has 258 beds (excluding Paediatrics and Day Case) and is one of the smallest rural district general hospitals in England (50). The prevalence rate of diabetes in older adults (aged 65 years and above) in 2017/2018 was found to be 24.4% in

Herefordshire, which is significantly higher than the West Midlands region (16.9%) and England (17.3%) (51).

Herefordshire is one of the most rural counties in England. Deprivation in rural areas may look different to that in urban areas. Access to services and housing are common challenges. In 2019 the income deprivation affecting older people index found that 11% (6100 people) of those aged over 60 were living in income deprived households and 12 lower support output areas are within the 25% most deprived in England(52). This has likely worsened since the cost of living crisis.

The scoping review undertaken revealed that that most studies published in the UK exploring or examining insulin errors are undertaken in large teaching NHS hospitals.

There has been recent recognition that rural, remote and coastal areas face different risks and exacerbations post COVID-19 pandemic (53). The challenges these areas face are often different to those in urban areas, for example a high proportion of vulnerable older adults, accessibility to services challenges, models of care that were designed for urban service delivery, staffing recruitment, retainment and training challenges. The National Institute for Health and Care Research (NIHR) has recognised there is a need for research to improve the quality and organization of healthcare in these areas recognising their differences to urban areas, where most research takes place(54).

7 SAMPLE AND RECRUITMENT

7.1 Eligibility Criteria

- Patients/their carers receiving care in the relevant organization study site designated wards
- Staff working in the relevant NHS organization study site

7.1.1 Inclusion criteria

Service user/Patient inclusion criteria

- Are able to give informed consent
- Age ≥ 65 years OR frailty identified/documented on admission
- Patient with a diagnosis of diabetes before their hospital admission and treated with insulin therapy before and during hospital stay
- History of hospital admission for major surgery with minimum 1 night hospital stay post-surgery at study site selected surgical wards: within the last 9 months.
- Fluent English speaker. Portuguese speaking patients can be included at interview stage

Family member/carer inclusion criteria

- Family members such as older/frail patients' children, siblings, spouses, friends or carers who speak and understood English and are involved in the usual diabetes care/insulin management of eligible patients may be considered for inclusion if invited by the patient/service user

NHS Staff inclusion criteria

- Clinical and non-clinical staff involved in the hospital care/transfer of care/insulin incident or safety review of older/frail adults with diabetes undergoing surgical admission at the study site designated wards
- Working full or part time at the study sites (for a minimum of 3 months)

7.1.2 Exclusion criteria

Service user/Patient exclusion criteria

- Unable to give informed consent
- Patients that don't have a formal diagnosis of diabetes prior to their hospital admission
- Patients with diabetes not treated with insulin therapy prior to and during their hospital stay
- Under age 65 years if absence of frailty on admission
- Unable to communicate effectively in English/Portuguese for interview stage
- Unable to communicate effectively and fluently in English for the workshops and co-design events
- Don't have history of hospital admission for major surgery with minimum 1 night hospital stay post-surgery within last 9 months at study site designated wards
- Patients undergoing day surgery without an overnight stay and patients who had a minor procedure will be excluded.
- History of hospital admission at the study sites but not for major surgical procedure and not admitted to the study site designated wards

Family member/carer exclusion criteria

- Patient does not invite/consent to family members or carers being involved
- Family member/Carer unwilling to participate

NHS staff exclusion criteria

- Clinical and non-clinical staff not involved in the hospital care/transfer of care/insulin incident or safety review of older/frail adults with diabetes undergoing surgical admission at the study site designated wards
- Employed at the study sites for less than 3 months
- Locum and agency staff
- For the non-participatory observations of incident review/safety meetings: staff who opt out

7.2 Sampling

Sampling will be purposive and include a number of pathways to ensure maximum inclusivity, further discussed in the Recruitment section.

7.2.1 Size of sample

The sample will aim to include up to 12-15 patients with diabetes/their carers and up to 25 members of NHS staff. The sample includes a larger number of NHS staff to represent the different roles in the interview phase and capture the range of experiences and perspectives of several different professional roles (eg. nurses of different grades & experience, medical/surgical workforce of different grades & experience, pharmacists, dietitians, medicine safety officer, governance and risk staff, management roles).

NB Carers/Family members are welcome to take part in this research, however their participation will be determined by the individual patients/service users who choose to take part. I am unable to specify at this stage how many carers may be involved, as it is up to the patients to invite their carers if they so wish. Anyone invited to accompany a patient will be eligible to participate and contribute to the co-design process based on their experience as a carer. The term carer will be defined by the patient i.e. whoever they choose to bring as their carer.

Numbers of participants in co-design studies vary depending on scale and number of sites but the numbers in this study are similar to other co-design studies when considering it is a single site study (55,56)Whilst initial numbers have been set, information power will be used throughout the study to evaluate sample size as study progresses (57). The concept of information power alludes to the more information pertinent to the study held within the sample, the lower the sample size needs to be, and vice versa (57). The purposive sampling and use of interview topic guides will help give information power to the sample, looking to achieve a robust set of perspectives on the problem being explored.

It is anticipated that the numbers of participants in the co-design events will be smaller, as there is likely to be some drop-out. For the workshops the plan is to invite all the patients who were interviewed. All NHS staff who are interviewed will be invited to express if they have an interest in being involved in the co-design process. Depending on interest, a sample of staff that is as representative as possible of different roles and experiences will be selected. Efforts will be taken to have as much as is feasible a similar number of patients/carers and staff and variety of staff roles represented in the co-design workshops noting the risk of power imbalances and of perspectives represented.

For the non-participatory observations of incident review/safety meetings:

A whole population sample of NHS staff present at the incident/safety review meetings will be observed. Attempts will be made to observe a minimum of 2 meetings, however this will be influenced by organizational and operational capacity to accommodate this.

7.2.2 Sampling technique

For the non-participatory observations of incident review/safety meetings:

Whole population sample of NHS staff present at the incident/safety review meetings will be observed.

For the interviews and workshops:
Purposive sampling will be used to aim to accommodate a range of views and experiences by recruiting staff from a range of different grades, professional roles, and experience of clinical and non-clinical staff involved in the hospital care/transfer of care/insulin incident or safety review of older/frail adults with diabetes undergoing surgical admission at the study site designated wards.
Purposive sampling amongst patients with diabetes/their carers who had a surgical admission at the study site designated wards will enable inclusion of people with different type of diabetes and insulin treatment, level of frailty, duration of hospital admission, reported insulin error occurrence or not.

If participants decide to withdraw from the study after one part of the study (eg. interviews) then attempts will be made to recruit additional participants if required.

7.3 Recruitment

7.3.1 Sample identification

For the non-participatory observations of incident review/safety meetings:

Observations will be on an opt-out basis as no data specific to any staff or service-user will be collected (further described in section 7.3.2). The focus of these observations is to have a sense of what things look like in practice regarding insulin error/insulin use data/safety review & learning and to understand processes and interactions, so that the context the staff and patients are in is understood ahead of the interviews and

co-design interactions with patients and staff. Observations will focus on type, amount and quality of interactions and organizational processes.

The lead researcher will liaise with the Principal Investigator and the Medicines Safety Officer at Hereford County Hospital (Wye Valley NHS Trust) about the incident review/safety meetings to observe. This will enable to have a presence at the meeting but not to collect data from individual patients or staff.

The observation sample will be the whole population of staff present at the meeting.

Posters to describe the project as well as photographs of the research team will be circulated beforehand. Posters will inform staff that the lead researcher will be carrying out observations and what the nature of those observations will be, and where they can get further information if they wish and how they can opt out.

For the interviews and workshops:

Service user/Patients with diabetes and their carers

Recruitment of patients/their carers will be both via clinicians working at the hospital sites and self-referral via a number of routes. Promotion of the study will be via clinicians, diabetes specialist team, service user involvement leads in the hospital site, posters in diabetes centres/clinics associated to the study site, and study sites social media. Healthwatch Herefordshire will also support with dissemination of study through their networks.

Clinicians/diabetes team in the hospital sites will be asked to identify potential participants and seek consent to be contacted by the research team.

Service user involvement leads will disseminate information about the study to service users, providing a telephone number and email address to self-refer if interested.

The study will be promoted through recruitment posters, service user involvement forums/groups and on social media.

The posters and social media will have brief information about the study and provide a telephone numbers and email address enabling relevant service users to self-refer if interested.

Once self-referral or consent to contact has been established, the lead researcher will make contact, provide potential participants with a brief overview of the study, and answer any questions.

If the individual wishes to be involved in the study, they will be sent a copy of the participant information sheet via post or email. This information sheet outlines the purpose and nature of the study, and the ethical safeguards regarding data protection and privacy. Potential participants will have at least 24 hours to consider whether they would like to be involved in the study.

If the individual would like to take part in the study, the lead researcher will liaise with them to arrange future meetings as appropriate to complete the consent process and give them the relevant information to read and consider.

Patients reasonable travel expenses to research activities will be reimbursed.

A £15 amazon voucher or One4all voucher will be provided to patients once as a thank-you for participation in the study. Light refreshments will be provided at the co-design events.

NB Carers/Family members are welcome to take part in this research, however their participation will be determined by the individual patients/service users who choose to take part. I am unable to specify at this stage how many carers may be involved, as it is up to the patients to invite their carers if they so wish. Anyone invited to accompany a patient will be eligible to participate and contribute to the co-design process based on their experience as a carer. The term carer will be defined by the patient i.e. whoever they choose to bring as their carer.

Staff Recruitment

The study will be promoted via recruitment posters at the hospital site via official communication methods (email, newsletter, trust's social media, physical posters).

Clinicians will also be recruited via team leaders, local diabetes team, word of mouth and email communications promoting the project and self-referral.

Once self-referral or verbal consent to contact has been established, the lead researcher will make contact, provide potential participants with a brief overview of the study, and answer any questions.

They will be given a participant information sheet and consent form and given sufficient time to make a decision, after which a written informed consent will be obtained if they decided to participate.

A £15 amazon voucher or One4all voucher will be provided once as a thank-you for participation in the study. Light Refreshments will be provided at the co-design events.

7.3.2 Consent

This study has varied forms of data collection (observations, interviews and co-design events) amongst different types of participants (patients, their carers and staff) and consent will be dealt with in different ways accordingly. Individual informed consent will be gained for interviews and co-design events. Observations will be on an opt-out basis as no data specific to any staff or service-user will be collected. This will be further described below.

Capacity for consent will be assessed in the following ways:

- understand the purpose and nature of the research
- understand what the research involves, its benefits (or lack of benefits), risks and burdens
- understand the alternatives to taking part
- be able to retain the information long enough to make an effective decision.
- be able to make a free choice
- be capable of making this particular decision at the time it needs to be made (though their capacity may fluctuate, and they may be capable of making some decisions but not others depending on their complexity)
- where participants are capable of consenting for themselves but are particularly susceptible to coercion, it is important to explain how their interests will be protected

Consent for the non-participatory observations of incident review/safety meetings:

- Observations will be on an opt-out basis as no data specific to any staff or service-user will be collected.
- The focus of these observations is to have a sense of what things look like in practice regarding insulin error/insulin use data/safety review & learning and to understand processes and

interactions, so that the context the staff and patients are in is understood ahead of the interviews and co-design interactions with patients and staff. Observations will focus on type, amount and quality of interactions and organizational processes.

- Overall site consent from the senior clinician (principal investigator) and the Medicines Safety Officer at NHS Trust will be gained; to enable to have a presence at the meeting but not to collect data from individual patients or staff.
- Prior to commencing the observations, information about the study will be discussed with the chair of the meeting(s) and where possible presented to staff at interprofessional meetings beforehand.
- Posters to describe the project as well as photographs of the research team will be circulated beforehand. Posters will inform staff that the lead researcher will be carrying out observations and what the nature of those observations will be, and where they can get further information if they wish and how they can opt out.
- An explanation of the project will be developed and used on arrival at the meeting.
- If any person at the meeting asks for observations not to be carried out in their vicinity, this will be adhered to immediately.
- If it is thought that observations are adversely affecting routine activities, they will be terminated immediately and recommenced at a time when they no longer adversely affect activities.
- Acknowledging the need for sensitivity, especially during non-participant observations, a pragmatic process approach to consent will be used, regularly checking that staff agree to being observed, and if anybody changes their mind and wishes to opt out this will be adhered to immediately and observations terminated.

Consent for the interviews and co-design events:

Written informed consent will be obtained from all participants prior to the participant undergoing any activities that are specifically for the purposes of the study.

Service-users may invite one carer to the research events. The lead researcher will give the service-user an information sheet and consent form for the carer, and will collect the carer's consent form on the day of the event/s that will be attended.

All identified potential participants will be given a copy of the participant information sheet (PIS) and they will be provided with adequate information and enough time to decide whether to participate. For patients, the clinician will assess the potential participant's ability to provide consent prior to being approached for the study.

A participant information sheet (appendixes staff and patient PIS) and consent form (appendixes staff and patient PIS) will explain the purpose, processes, activities, potential risk and benefits of participation. It will be emphasised that participation is voluntary and that they can withdraw their consent without having to explain why, and will be reassured that their decision will not affect the care that they will receive.

8 ETHICAL AND REGULATORY CONSIDERATIONS

No aspects of the study will commence until ethics, HRA and R&D approvals have been received.
Good Clinical Practice Guidelines will be observed throughout all stages of this project.

8.1 Assessment and management of risk

Consent is an ongoing process.

8.1.1 Respecting participant autonomy

Written informed consent will be obtained from all participants participating in interviews and co-design events; they will be provided with adequate information and enough time to decide whether to participate. The clinician will assess the potential participant's ability to provide consent prior to being approached for the study.

A patient information sheet and consent form will explain the purpose, processes, activities, potential risk and benefits of participation. It will be emphasised that participation is voluntary and that they can withdraw their consent without having to explain why, and will be reassured that their decision will not affect the care that they will receive.

Given the nature of the patient population included in this study is going to have people with frailty, to assist people to be able to participate if they want to, there are 2 versions of the consent form. One is a written standard consent form and the other is a more visual and accessible one for patients in such circumstances. This would enable more equitable recruitment of people to the study. The visual consent form was adapted from an accessible consent form used in the CREATE co-design study with patients in a stroke unit (58) The importance of having an accessible consent form was voiced by the patient group consulted during the preparation of the study documentation.

8.1.2. Inadvertent disruption of practice

All study activities will be planned in such a way that normal patient appointments and procedures would not be affected. As much as possible, co-design events will be conducted at a time that is convenient for patients and staff. The lead researcher will liaise with the clinical teams to assess appropriate timing for data collection. The lead researcher will familiarise herself with the ward environments and processes.

Visits of ward environments prior to start of the study will be arranged.

8.1.3. Purpose and Design

Using a co-design approach, patients will have a 'voice' in developing the intervention and become co-designers using their experiences. However, in co-design workshops, patients may feel less empowered due to traditional relationships between healthcare professionals and patients, where healthcare professionals tend to have more power. Further, participants may not be accustomed to working together. To overcome these barriers, an experienced facilitator will support the co-design events to encourage listening and enable speaking up.

8.1.4. Risk to researcher

Study activities will be conducted in the hospital site or if the patient participant is unable to come to hospital, the lead researcher will offer to conduct the interview virtually. This way potential safety risks to the researcher are minimised.

8.1.5. Protecting participants and the public from harm

The target patient population may have experienced difficult experiences during their hospital admission and they may still be recovering following their hospital admission.

This could be a difficult time for patient participants. The lead researcher is a diabetes specialist nurse and has experience of supporting patients during this point in their diabetes management journey.

The lead researcher will ensure sensitivity when approaching potential participants and make the necessary referrals to the team if needed at any point within the study.

Participants will be encouraged to share their experiences without naming names. Sensitive or upsetting topics may be brought up in interviews and/or group discussions. If the participant becomes distressed or upset at any point during study activities, the researcher will offer to stop the session and will only recommence if and when and the participant is ready to do so. There will be no pressure on the participant to continue. Constant assessment will be made and participants will be asked if they are still willing to go ahead with participation. If applicable, they will be referred back to their clinical team (with their consent) for assessment and possible referral to the local services available for further psychological and emotional support.

If the participant shows any evidence of suicidal thoughts or ideation, their clinical team will be informed immediately as guided by the researcher's professional conduct and standards i.e. Nursing and Midwifery Council Code (2015).

In the unlikely event that participants disclose an incident that may cause direct harm to any member of the public including themselves, the researcher will report this, despite the effects it may have on the study.

If the individual is a staff member, normal escalation procedures will be followed. If the disclosure is from a patient, the primary supervisor will be notified immediately to decide on the appropriate course of action. However, the researcher will also act within the boundaries of her professional code of conducts/standards and has a duty to protect the public.

Participants will be informed through the participant information sheet about the type of information that the researcher would be obliged report. Participants will be informed that their study participation is completely voluntary.

There may be some inconvenience to participants when attending study activities. As much as possible, the lead researcher will arrange study schedules to minimise inconvenience with regards to time and travel.

8.1.6. Inclusion/Exclusion

Written participant information sheets will only be in English because this translation activity is not covered in the funding.

Written participant information sheets will only be in English because this translation activity is not covered in the funding.

Given the lead researcher is also fluent in Portuguese, it is possible for interviews to also be conducted in Portuguese, if this facilitates participation of Portuguese speaking patients.

Due to the complex nature of feedback and co-design events, patients who are not fluent in English will be excluded. The presence of a translator may influence spontaneity and clarity of discussions. Nonetheless, the design of the study will consider the patient's 'voice' through observations and interviews.

8.2 Research Ethics Committee (REC) and other Regulatory review & reports

Before the start of the study, a favourable opinion will be sought from a REC for the study protocol, informed consent forms and other relevant documents e.g. advertisements.

- Substantial amendments that require review by NHS REC will not be implemented until that review is in place and other mechanisms are in place to implement at site.
- All correspondence with the REC will be retained.

- It is the Chief Investigator's responsibility to produce the annual reports as required.
- The Chief Investigator will notify the REC of the end of the study.
- An annual progress report (APR) will be submitted to the REC within 30 days of the anniversary date on which the favourable opinion was given, and annually until the study is declared ended.
- If the study is ended prematurely, the Chief Investigator will notify the REC, including the reasons for the premature termination.
- Within one year after the end of the study, the Chief Investigator will submit a final report with the results, including any publications/abstracts, to the REC.

8.2.1 Regulatory Review & Compliance

- Before the site can enrol patients into the study, the Chief Investigator/Principal Investigator or designee will ensure that appropriate approvals from participating organisations are in place. Specific arrangements on how to gain approval from participating organisations are in place and comply with the relevant guidance.
- For any amendment to the study, the Chief Investigator or designee, in agreement with the sponsor will submit information to the appropriate body in order for them to issue approval for the amendment. The Chief Investigator or designee will work with sites (R&D departments at NHS sites as well as the study delivery team) so they can put the necessary arrangements in place to implement the amendment to confirm their support for the study as amended.

8.2.2 Amendments

If a substantial amendment to the REC application or the supporting documents is required, the research team will submit a notice of amendment to the REC for consideration and to relevant NHS site R&D department.

The lead researcher and the chief investigator will decide whether to amend the protocol and whether an amendment is substantial using HRA definitions.

The amendment history will be tracked using protocol version control.

Changes will be communicated to the REC and NHS site R&D using their established processes, for example the IRAS substantial amendments form.

8.3 Peer review

The study protocol has been reviewed by the lead researcher's supervisory team at King's College London. Dr Sara Donetto from Brighton and Sussex Medical School kindly reviewed the protocol and offered comments and feedback from a methodological perspective.

8.4 Patient & Public Involvement

As patients are key stakeholders in the use of insulin in hospital, patient involvement has been sought prior to this application and will continue to be a key component of this study.

The following PPI has been/will be sought for this study:

Acceptability of the research

In the years leading up to the development of this study, the lead researcher who is also a nurse working in the NHS has had multiple informal conversations with members of staff and people with diabetes/their carers about the complex problem of insulin errors and insulin safety in hospital. Patients welcome the idea of further research into this area, feeling more needs to be done to improve patient safety and

patient trust in the healthcare professionals looking after them. NHS staff also feel insulin safety is important and that better ways to support and engage clinicians are needed.

Whilst undertaking the NIHR 'Silver Scholar' pre-doctoral award, the research topic of insulin errors and insulin safety in hospital was presented to a group of people with diabetes and their carers at a local Diabetes UK AGM to gage people's thoughts and ideas around acceptability of research in this area. Overwhelmingly the audience felt this should be a research priority, with many voicing experiences where they felt insulin safety in hospital needed to significantly improve. More recently, engagement with a local patients with diabetes group once more confirmed that patients would value more research into hospital insulin safety, and this is something they consider very important.

These shared conversations over the years, informed the decision for taking a co-design approach, where services are created by patients and staff for patients and staff, and views of those receiving care are also included.

Design of the research

Informal feedback from a patient with diabetes who is also a NHS worker

A local patients with diabetes group reviewed and provided feedback on the plain summary for the project, recruitment posters, PIS and consent forms. They particularly thought it was important to have an accessible consent form. They also fully supported the project and thought it was very important; many of them related it to previous experiences they had had in hospital and welcomed the thought of more research involving patients in this important area.

They felt the facilitating team at the co-design events would be really important to ensure people felt safe and comfortable to discuss ideas. They have had experience of events facilitated by the facilitators this project will be using and felt that those individuals are the right ones to facilitate patient and staff co-design events.

Some of the discussions at the group informed the development of the interview topic guides, in particular about exploration of self-management of insulin in hospital

The project was presented to Healthwatch Herefordshire team who agreed with importance of project and felt it resonated with issues and concerns voiced by people with diabetes which they had identified in their own projects and engagement with patients in the local community. Local Healthwatch agreed to support with the following:

- Reviewing study documentation through their reading panel
- Dissemination of call to recruitment
- Offer pathways into particular user groups/stakeholders for consultation and dissemination purposes
- Dissemination of findings through their networks
- Highlight possible areas of collaboration with other projects

Undertaking of research/Analysis of research/Dissemination of findings

By using a co-design approach, patients and staff involved in the feedback/co-design workshops will have a 'voice' in selecting the key priorities/areas the intervention to develop will address.

If funding application is successful Graphic designer input will be commissioned to develop an infographic of patient priorities in hospital insulin safety as told by patients. This is in response to PPI engagement which indicated the need for clear dissemination (including graphic/visuals) of patient voice accessible to a wider audience than the academic one.

8.5 Protocol compliance

The lead researcher will ensure that the protocol is followed.

Accidental protocol deviations can happen at any time. They must be adequately documented on the relevant forms and reported to the Chief Investigator and Sponsor immediately.

Deviations from the protocol which are found to frequently recur are not acceptable, will require immediate action and could potentially be classified as a serious breach.

8.6 Data protection and patient confidentiality

The sponsor and data controller for this project will be King's College London (KCL). The collection, storage and disposal of the data during this study will be kept following the UK Data Protection Act 2018 and General Data Protection Regulation (GDPR). Participants can provide their consent for the use of their personal data in this study by completing the consent forms provided to them. The lead researcher will be the data custodian. To reduce the risk of identification, identifiable and anonymised data will be stored in separate locations.

Participants will be informed about personal data that will be collected and the purpose of collecting these. Each participant will be given a unique participant identification number at consent stage. Only the lead researcher will have access to these data and ensure that real identities of participants cannot be connected with allocated study identifiers. Identity of participants will be kept confidential, access to records will be limited to researchers directly involved in the study, all paper and electronic files will be identified by a unique study number.

All the participants personal data, consent, qualitative interview and workshop recordings will be encrypted before they are uploaded to a secure and encrypted cloud storage area provided by KCL and will only be accessible by the study team members. Once the study has been completed the database will be kept for seven years after the study completion which is in accordance with KCL policy and will then be destroyed by deleting it from the encrypted and password protected file sharing cloud.

Interviews and feedback/co-design events will be recorded on an encrypted digital audio recorder and will be securely transferred to a King's College London password protected server. They will either be transcribed by the lead researcher on a King's College London password protected server, or may also be uploaded in encrypted form to a professional transcription website. Transcripts will be downloaded from this website to the King's College London password protected server. Transcripts will be anonymised and uploaded to NVivo application (QSR International) software within a King's College London password protected server. Data analysis will be conducted by the lead researcher and will be scrutinised by the supervisory team.

All qualitative observational data (e.g. observations of incident review meetings and co-design workshops) will be completed in either paper form as field notes or on a data collection tool on tablet, either at the participating hospital site or at the co-design workshops.

The field notes will not contain any identifiable information about specific participants as the data will be looking broadly at activities, interactions, group dynamics, rather than specific people. The data will be

manually uploaded into the qualitative analysis package NVivo application (QSR International) software within a King's College London password protected server, then analysed by the study lead researcher and will be scrutinised by the supervisory team.

Data will not be shared between the research team via email unless it is transferred as encrypted data. No data will be stored on personal computers, USB drives, CD or portable hard drives.

Identifiable information divulged in observations, interviews or co-design events will be removed at the point of transcription, electronic recording of interviews will be erased after data analysis.

Procedures for anonymisation:

Each participant will be given a unique participant identification number at consent stage. Interview data and feedback/co-design event data will use this number and not the participant's name. Interview data and feedback/co-design event data will be transcribed and anonymised to ensure participant's name, location or any other identifying information is removed. Each transcript will be given the participant identification number. Only study team members will have access to study data.

Identifiable data will be kept separately on a secure server on a NHS hospital password protected laptop and/or in a locked cabinet within the diabetes team offices at Hereford County Hospital, accessible by the lead researcher only. The offices are passcode protected and shared by the diabetes team.

The ID code break sheet linking identifiable data to the unique study identification number code of each participant will be kept separate to the research data, in a locked cabinet within the diabetes team offices at Hereford County Hospital, accessible by the lead researcher only. The offices are passcode protected and shared by the diabetes team.

Anonymised quotes used in any publications and/or conference presentations will be scrutinised to ensure that a person cannot be identified from the combination of quotes.
All contact details will be destroyed on completion of the study. Audio recordings will be deleted once analysis has been completed.

8.7 Indemnity

As the lead Sponsor King's College London Insurance will cover potential legal liability for harm to participants arising from the design of the study.

Wye Valley NHS Trust site will be covered by the NHS indemnity scheme.

8.8 Safety & Adverse Events Reporting

The researchers do not anticipate any serious adverse events (SAEs) occurring as a result of study participation.

Guided by the Health Research Authority Safety Reporting procedures, SAEs in this study are defined as cases that have either (1) related to the study *i.e.* resulted from administration of any of the research procedures or (2) unexpected *i.e.* not listed in the protocol as an expected occurrence.

Where KCL is the Sponsor and an SAE occurs that does not require immediate reporting, this SAE should be reported in the Annual Progress Report where applicable (see Progress Reporting section below, appendix 24) and copied to the RGO, alongside any AEs that occur that are not classified as 'serious'.

All adverse events that are to be reported to the RGO must be signed and dated and completed by the Chief Investigator (N.B. Data breaches are also classified as SAEs).

Reports of related and unexpected SAEs will be submitted to the Main NHS/ HSC REC within 15 days of the Chief Investigator becoming aware of the event, using the appropriate template. The form will be completed in typescript and signed by the Chief Investigator. The main REC will acknowledge receipt of safety reports within 30 days. A copy of the SAE notification and acknowledgement receipt will also be sent to the RGO.

(HRA's guidance on adverse events: https://www.hra.nhs.uk/approvals-amendments/managing-your-approval/safety-reporting/)

Further Information with regards to Safety Reporting in Non-CTIMP Research can be found in appendix 24.

8.9 Access to the final study dataset

The lead researcher and study supervisors will have access to the full dataset.

8.10 End of Study Reporting

8.10.1 End of Study Declaration

The end of the study will be declared to the REC that gave a favourable opinion (as per the above Regulatory Approvals section) within 90 days of the study ending.

An end of study declaration form template can be found on the HRAs webpages: https://www.hra.nhs.uk/approvals-amendments/managing-your-approval/ending-your-project/

8.10.2 End of Study Reporting

The end of the study report will be submitted to the REC that gave a favourable opinion (as per the above Regulatory Approvals section) within 12 months of the study ending, using the following form:

https://www.hra.nhs.uk/approvals-amendments/managing-your-approval/ending-your-project/final-report-form/

For further information about the final report, see the HRA's questions and answers page.

9 DISSEMINATION POLICY

9.1 Dissemination policy

A wide range of dissemination methods will be used to ensure the project and emerging research findings are shared with relevant audiences: patients and the public, research community, healthcare professionals and policy makers. Some of these include:

- A study final report will be prepared on completion of the study and analysis of the data and circulated to patients and staff involved in the study.
- Presentation of results at local trusts research events or publications

- Liaison with Healthwatch Herefordshire regarding dissemination of findings outputs through their networks.
- Publication of results in peer reviewed journals/posters for example:
    - Findings of interviews with patients and HCP
    - Design and development of conceptual model of an intervention to be developed using a co-design approach
- Presentation of results at conferences and seminars and other public engagement activities

The funding body will be acknowledged within any study publications.

The full study report and data analysis including anonymised quotations will be made publicly available once the PhD thesis which this study is a part of is published.

9.2 Authorship eligibility guidelines and any intended use of professional writers

All study team members who make a substantive contribution to reading and writing the final report will be granted authorship on the final study report.

10 REFERENCES

1. Persaud SJ, Jones PM. Physiology of Glucose Homeostasis. In: Wass J, Arlt W, Semple R, editors. Oxford Textbook of Endocrinology and Diabetes 3e. 3rd ed. Oxford University Press; 2022. p. 1917–22.
2. Diabetes UK. Diabetes statistics | Professionals | Diabetes UK [Internet]. 2020 [cited 2021 Nov 25]. Available from: https://www.diabetes.org.uk/professionals/position-statements-reports/statistics
3. World Health Organization. Classification of diabetes mellitus 2019 [Internet]. 2019 [cited 2022 Mar 2]. Available from: https://www.who.int/publications/i/item/classification-of-diabetes-mellitus
4. Ramachandran A, Chamukuttan S, Nanditha A. Classification and Diagnosis of Diabetes. In: Holt RIG, Cockram C, Flyvberg A, Goldstein B, editors. Textbook of Diabetes [Internet]. 5th ed. John Willey and Sons; 2017 [cited 2022 Mar 2]. p. 23–8. Available from: https://ebookcentral.proquest.com/lib/kcl/reader.action?docID=4769056
5. National Institute for Health and Care Excellence. NICE Guideline NG 17: Type 1 diabetes in adults: diagnosis and management [Internet]. Last updated 17 August 2022. 2015 [cited 2022 Mar 16]. Available from: https://www.nice.org.uk/guidance/ng17
6. National Institute for Health and Care Excellence. NICE Guideline NG 28 Type 2 diabetes in adults: management | Guidance | NICE [Internet]. Last updated 29 June 2022. 2015 [cited 2022 Mar 16]. Available from: https://www.nice.org.uk/guidance/ng28
7. Choudhary P, Jacob P. Insulins and Insulin Delivery Devices. In: Wass J, Wiebke A, Semple R, editors. Oxford Textbook of Endocrinology and Diabetes 3e. 3rd ed. Oxford University Press; 2022. p. 1978–86.
8. Gough S, Narendran P. Insulin and Insulin treatment. In: Holt R, Cockram C, Flyvbjerg A, Goldstein B, editors. Textbook of Diabetes [Internet]. 5th ed. John Wiley & Sons, Ltd; 2017 [cited 2022 Mar 16]. p. 401–13. Available from: https://ebookcentral.proquest.com/lib/kcl/reader.action?docID=4769056
9. Institute for Safe Medication Practices (ISMP). 2017 ISMP Guidelines for Optimizing Safe Subcutaneous Insulin Use in Adults [Internet]. 2017 [cited 2021 Nov 4]. Available from: https://www.ismp.org/sites/default/files/attachments/2017-11/ISMP138-Insulin Guideline-051517-2-WEB.pdf

10. World Health Organization. Medication safety in transitions of care [Internet]. 2019 [cited 2021 Nov 29]. Available from: https://www.who.int/publications/i/item/WHO-UHC-SDS-2019.9

11. Care Quality Commission. Medicines in health and adult social care Learning from risks and sharing good practice for better outcomes [Internet]. 2019 [cited 2022 Mar 23]. Available from: https://www.cqc.org.uk/sites/default/files/20190605_medicines_in_health_and_adult_social_care_report.pdf

12. Bain A, Hasan SS, Babar ZUD. Interventions to improve insulin prescribing practice for people with diabetes in hospital: a systematic review. Diabet Med. 2019 Aug 1;36(8):948–60.

13. NHS Digital. National Diabetes Inpatient Audit England, 2019. 2020.

14. Braithwaite J, Clay-Williams R, Nugus P, Plumb J. Healthcare as a complex adaptive system. In: Hollnagel E, Braithwaite J, Wears RL, editors. Resilient Healthcare. Ashgate Publishing Ltd; 2013. p. 57–75.

15. Rayman G. Management of the Inpatient with Diabetes Mellitus. In: Wass J, Wiebke A, Semple R, editors. Oxford Textbook of Endocrinology and Diabetes Third Edition [Internet]. 3rd ed. Oxford University Press; 2022 [cited 2022 Mar 24]. p. 1–7. Available from: http://books.google.com/books?hl=en&lr=&id=aT1Z84G5S0sC&pgis=1

16. Rousseau M-P, Naud A-S, Leblond J, Cossette B, Grondin F, Lanthier L, et al. An interprofessional qualitative study of barriers and potential solutions for the safe use of insulin in the hospital setting. Can J Diabetes [Internet]. 2014;38(2):85–9. Available from: http://www.elsevier.com/wps/find/journaldescription.cws_home/727619/descriptio

17. Hamid T, Harper L, Rose S, Petkar S, Fienman R, Athar SM, et al. Prescription errors in the national health services, time to change practice. Scott Med J. 2016;61(1):1–6.

18. Al-Yassin A, Al-Khaja A, Jichi F, Clarke C, Lisk C, Katz JR. Introducing a diabetes e-learning module: A means of improving junior doctors' confidence and ability in managing inpatients with diabetes. Pract Diabetes. 2013;30(3).

19. WalkerCA, Osborne CA, Burmiston S, Thomas S. Use of failure severity and frequency to reduce insulin risk in secondary care. In 2011. p. Clinical Pharmacist vol 3 (4).

20. Wong VW, Ho A, Fiakos E, Lau NS, Russell H. Introduction of New South Wales adult subcutaneous insulin- prescribing chart in a tertiary hospital : its impact on inpatient glycaemic control. 2016;1323–8.

21. Rajendran R, Kerry C, Round R, Barker S, Scott A, Rayman G. Short Report : Educational and Psychological Issues Impact of the Diabetes Inpatient Care and Education (DICE) project and the DICE Care Pathway on patient outcomes and trainee doctor ' s knowledge and confidence. 2015;920–4.

22. Kelly NAA, Brandom KG, Mattick KL. Improving preparedness of medical students and junior doctors to manage patients with diabetes. BMJ Open Diabetes Res Care. 2015;3(1):e000116.

23. Ward, S.; Wasson G. Improving preparedness of junior doctors to manage patients with diabetes: a pilot educational programme from ward-based pharmacists. Br J Diabetes. 2017;17:152–5.

24. Bain A, Silcock J, Kavanagh S, Quinn G, Fonseca I. Improving the quality of insulin prescribing for people with diabetes being discharged from hospital. BMJ open Qual [Internet]. 2019;8(3):e000655. Available from: http://ovidsp.ovid.com/ovidweb.cgi?T=JS&PAGE=reference&D=pmnm4&NEWS=N&AN=31523740

25. Johnston P, Newland-Jones P. Insulin self-Administration: Development of an assessment tool to empower hospital inpatients with diabetes. J Diabetes Nurs. 2017;21(5):174–8.

26. Primary Care Diabetes Society. “Six Steps to Insulin Safety” e-learning module [Internet]. 2017 [cited 2021 Nov 4]. Available from: https://diabetesonthenet.com/diabetes-primary-care/six-steps-to-insulin-safety-e-learning-module-highlighted-by-nice/

27. Diabetes UK. Improving insulin safety in hospital. Diabetes Update Autumn 2017. 2017;20–4.

28. Diabetes UK. Making hospitals safe for people with diabetes [Internet]. 2018 [cited 2021 Nov 25]. Available from: https://www.diabetes.org.uk/resources-s3/2018-10/Making Hospitals safe for people with diabetes_FINAL.pdf

29. Snow, R; Humphrey, C; Sandall J. What happens when patients know more than their doctors? Experiences of health interactions after diabetes patient education: a qualitative patient-led study. BMJ Open Diabetes Res Care [Internet]. 2013;3(11). Available from: https://bmjopen.bmj.com/content/3/11/e003583

30. Kwon S, Thompson R, Dellinger P, Yanez D, Farrohki E, Flum D. Importance of Perioperative Glycemic Control in General Surgery: A Report From the Surgical Care and Outcomes Assessment Program. 2012 [cited 2022 May 8]; Available from: http://www.scoap.org/

31. Centre for Perioperative Care. Guideline for Perioperative Care for People with Diabetes Mellitus Undergoing Elective and Emergency Surgery Guideline for Perioperative Care for People with Diabetes Mellitus Undergoing Elective and Emergency Surgery Diabetes Guideline Working Group [Internet]. 2022 [cited 2022 May 8]. Available from: https://cpoc.org.uk/sites/cpoc/files/documents/2023-02/CPOC-Diabetes-Guideline-Updated2022_0.pdf

32. Ata A, Lee J, Bestle SL, Desemone J, Stain SC. Postoperative Hyperglycemia and Surgical Site Infection in General Surgery Patients. Arch Surg [Internet]. 2010 Sep 1 [cited 2022 May 8];145(9):858–64. Available from: https://jamanetwork.com/journals/jamasurgery/fullarticle/406267

33. Gunst J, Van den Berghe G. Care of Diabetes in ICU and Perisurgery. In: Wass J, Arlt W, Semple R, editors. Oxford Textbook of Endocrinology and Diabetes 3e. 3rd ed. Oxford University Press; 2022. p. 2090–4.

34. The National Confidential Enquiry into Patient Outcomes and Death. Highs and Lows: a review of the quality of care provided to patients over the age of 16 who had diabetes and underwent a surgical procedure [Internet]. 2018 [cited 2022 May 8]. Available from: www.hqip.org.uk/national-programmes

35. Rayman G, Kar P. Diabetes GIRFT Programme National Specialty Report. 2020.

36. NHS Digital. National Diabetes Inpatient Audit: Harms 2020 [Internet]. 2021 [cited 2022 May 8]. Available from: http://digital.nhs.uk/pubs/nadia-harms2020

37. Abdelhafiz AH, Sinclair AJ. Diabetes in Old Age. In: Holt RI, Cockram C, Flyvberg A, Goldstein BJ, editors. Textbook of Diabetes [Internet]. 5th ed. John Wiley & Sons Inc; 2017 [cited 2022 Apr 3]. p. 939–52. Available from: https://ebookcentral.proquest.com/lib/kcl/reader.action?docID=4769056

38. Joint British Diabetes Societies for Inpatient Care. Inpatient Care of the Frail Older Adult with Diabetes [Internet]. 2019 [cited 2022 Mar 29]. Available from: https://abcd.care/joint-british-diabetes-societies-jbds-inpatient-care-group

39. Hollnagel E, Wears RL, Braithwaite J. From Safety-I to Safety-II: A White Paper. 2015;

40. National Health Service England. Patient safety response learning toolkit [Internet]. 2022. Available from: https://www.england.nhs.uk/publication/patient-safety-learning-response-toolkit/

41. Skivington K, Matthews L, Simpson SA, Craig P, Baird J, Blazeby JM, et al. Framework for the development and evaluation of complex interventions: gap analysis, workshop and consultation-

informed update [Internet]. Vol. 25, NIHR Health Technology Assessment. 2021 Sep. Available from: https://www.journalslibrary.nihr.ac.uk/hta/hta25570

42. O'Cathain A, Croot L, Duncan E, Rousseau N, Sworn K, Turner KM, et al. Guidance on how to develop complex interventions to improve health and healthcare. BMJ Open [Internet]. 2019 Aug 1 [cited 2021 Sep 22];9(8):e029954. Available from: https://bmjopen.bmj.com/content/9/8/e029954

43. Ku B, Lupton E. Health Design Thinking. New York: Cooper Hewitt; 2020. 215 p.

44. IDEO. The Field Guide to Human-Centered Design [Internet]. Canada; 2015. Available from: https://www.ideo.com/post/design-kit

45. Raynor DK, Ismail H, Blenkinsopp A, Fylan B, Armitage G, Silcock J. Experience-based co-design-Adapting the method for a researcher-initiated study in a multi-site setting. Health Expect [Internet]. 2020 Jun 1 [cited 2022 Jan 26];23(3):562–70. Available from: https://pubmed.ncbi.nlm.nih.gov/32045087/

46. Anderson JE, Watt AJ. Using Safety-II and resilient healthcare principles to learn from Never Events. Int J Qual Heal Care. 2020;32(3):196–203.

47. IDEO. From Ideas to Action toolkit. Innovate Your Innovation Process. 2022.

48. Ritchie J, Spencer L. Qualitative data analysis for applied policy research. In: Bryman A, Burgess B, editors. Analyzing Qualitative Data. Taylor & Francis Group; 1994. p. 173–94.

49. Locock L, Robert G, Boaz A, Vougioukalou S, Shuldham C, Fielden J, et al. Testing accelerated experience-based co-design: a qualitative study of using a national archive of patient experience narrative interviews to promote rapid patient-centred service improvement. Heal Serv Deliv Res. 2014 Feb 25;2(4):1–122.

50. Wye Valley NHS Trust. About Wye Valley NHS Trust [Internet]. online. 2022. Available from: https://www.wyevalley.nhs.uk/work-with-us/about-wye-valley-nhs-trust.aspx

51. Understanding Herefordshire. Diabetes [Internet]. online. 2022. Available from: https://understanding.herefordshire.gov.uk/health/topics-relating-to-health-conditions/diabetes/#:~:text=The highest prevalence was recorded in North and,Midlands NHS Region %2816.9%25%29 and in England %2817.3%25%29.

52. Understanding Herefordshire. Income deprivation - Income deprivation affecting older people sub-domain [Internet]. online. 2020. Available from: https://understanding.herefordshire.gov.uk/inequalities/index-of-multiple-deprivation-imd/income-deprivation-income-deprivation-affecting-older-people-sub-domain/#:~:text=There were approximately 6%2C100 older people living in,Hereford%2C three in Leomins

53. Palmer B, Rolewicz L. Rural, remote and at risk: Why rural health services face a steep climb to recovery from Covid-19 [Internet]. Nuffield Trust Report. 2020. Available from: https://www.nuffieldtrust.org.uk/research/rural-remote-and-at-risk

54. National Institute for Health and Care Research. 23/91 Improving the Organisation and Quality of Health and Social Care Services in Rural and Coastal Areas of the UK - supporting information [Internet]. 2023. Available from: https://www.nihr.ac.uk/documents/2391-improving-the-organisation-and-quality-of-health-and-social-care-services-in-rural-and-coastal-areas-of-the-uk-supporting-information/33649

55. Due-Christensen M, Joensen LE, Sarre S, Romanczuk E, Wad JL, Forde R, et al. A co-design study to develop supportive interventions to improve psychological and social adaptation among adults with new-onset type 1 diabetes in Denmark and the UK. BMJ Open [Internet]. 2021 Nov 2 [cited 2022 Apr 15];11(11). Available from: https://pubmed.ncbi.nlm.nih.gov/34728449/

56. Hjelmfors L, Strömberg A, Friedrichsen M, Sandgren A, Mårtensson J, Jaarsma T. Using co-

design to develop an intervention to improve communication about the heart failure trajectory and end-of-life care. BMC Palliat Care [Internet]. 2018 Jun 11 [cited 2021 Dec 14];17(1):1–10. Available from: https://bmcpalliatcare.biomedcentral.com/articles/10.1186/s12904-018-0340-2

57. Malterud K, Siersma VD, Guassora AD. Sample size in qualitative interviews: guided by information power. Qual Health Res. 2016;26(13):1753–60.

58. Jones F, Gombert-Waldron K, Honey S, Cloud G, Harris R, Macdonald A, et al. Using co-production to increase activity in acute stroke units: the CREATE mixed-methods study. Heal Serv Deliv Res. 2020;8(35):1–136.

11. APPENDICIES

11.1 Appendix 1-23 Required documentation

Appendix 1 Participant Information Sheet (PIS) for service users/patients

Appendix 2 Participant Information Sheet (PIS) for NHS Staff

Appendix 3 Consent form for service user patient interviews

Appendix 4 Accessible Consent form for service user patient interviews

Appendix 5 Consent form for service user patient workshop

Appendix 6 Accessible Consent form for service user patient workshop

Appendix 7 Consent form for integrated service user and staff workshop

Appendix 8 Accessible Consent form for integrated service user and staff workshop

Appendix 9 Consent form for NHS staff interviews

Appendix 10 Consent form for NHS staff workshop

Appendix 11 Consent to contact form

Appendix 12 Patient demographics form

Appendix 13 NHS Staff demographics form

Appendix 14 Interview topic guide patients

Appendix 15 Interview topic guide NHS Staff

Appendix 16 Letter to GP

Appendix 17 Recruitment Opt out poster for observations

Appendix 18 Recruitment poster NHS Staff

Appendix 19 Recruitment poster Service users

Appendix 20 Invitation letter service user workshop events

Appendix 21 Invitation letter staff workshop events

Appendix 22 Student/Lead researcher CV (CLF)

Appendix 23 Chief Investigator CV (KW)

11.2 Appendix 24 Information with regards to Safety Reporting in Non-CTIMP Research

	Who	When	How	To Whom
**SAE**	Chief Investigator	Report to Sponsor within 24 hours of learning of the event<br><br>Report to the MREC within 15 days of learning of the event	SAE Report form for Non-CTIMPs, available from NRES website.	Sponsor and MREC
**Urgent Safety Measures**	Chief Investigator	Contact the Sponsor Immediately<br><br>MREC to be notified Within 3 days	By phone/email<br><br>Initial notification must set out the reasons for the urgent safety measures and the plan for further action.<br><br>Where required, Substantial amendment should be submitted as soon as it is possible to do so.	Main REC and Sponsor<br><br>MREC will aim to give a formal opinion on the substantial amendment within 28 calendar days but will give an opinion in no more than 35 days.
**Minor Protocol deviations or GCP non-compliance**	Chief Investigator	Contact the Sponsor as soon as possible after learning of the event	By email using the file note template, protocol deviation log and/or file note log templates	Sponsor<br><br>Voluntary notification to REC manager and to breaches@hra.nhs.uk for information
**Serious Breaches**	Chief Investigator	Contact the Sponsor immediately<br><br>MREC to be notified within 7 days of Sponsor notification	By email including details of when the breach occurred, the location, who was involved, the outcome and any information given to participants. An explanation should be given, and the REC informed what further action the sponsor plans to take.	Main REC and Sponsor<br><br>Reports provided may be referred to the Health Research Authority breaches@hra.nhs.uk for consideration by the Main REC
**Progress Reports**	Chief Investigator	Annually (starting 12 months after the date of favourable opinion)	Annual Progress Report Form (non-CTIMPs) available from the NRES website	Main REC

**Declaration of the conclusion or early termination of the study**	Chief Investigator	Within 90 days (conclusion)<br><br>Within 15 days (early termination)<br><br>*The end of study should be defined in the protocol*	End of Study Declaration form available from the NRES website	Main REC with a copy to be sent to the sponsor
**Summary of final Report**	Chief Investigator	Within one year of conclusion of the Research	No Standard Format However, the following Information should be included:-<br>Where the study has met its objectives, the main findings and arrangements for publication or dissemination including feedback to participants	Main REC with a copy to be sent to the sponsor

13.2 Amendment History

Amendment No.	Protocol version no.	Date issued	Author(s) of changes	Details of changes made
